# Long-range allostery mediates cooperative adenine nucleotide binding by the Ski2-like RNA helicase Brr2

**DOI:** 10.1016/j.jbc.2021.100829

**Published:** 2021-05-26

**Authors:** Eva Absmeier, Karen Vester, Tahereh Ghane, Dmitry Burakovskiy, Pohl Milon, Petra Imhof, Marina V. Rodnina, Karine F. Santos, Markus C. Wahl

**Affiliations:** 1 Structural Biochemistry, Freie Universität Berlin, Berlin, Germany; 2 Computational Biophysics, Freie Universität Berlin, Berlin, Germany; 3 Department of Physical Biochemistry, Max Planck Institute for Biophysical Chemistry, Göttingen, Germany; 4 Centre for Research and Innovation, Health Sciences Faculty, Universidad Peruana de Ciencias Aplicadas, Lima, Peru; 5 Macromolecular Crystallography, Helmholtz-Zentrum Berlin für Materialien und Energie, Berlin, Germany

**Keywords:** allosteric regulation, enzyme kinetics, intramolecular regulation, pre-mRNA splicing, protein conformation, RNA helicase, superfamily 2 helicase, BAC, bacterial artificial chromosome, CC, C-terminal cassette, DSF, differential scanning fluorimetry, DTT, dithiothreitol, FRET, fluorescence resonance energy transfer, h, human, HB, helical bundle domain, HLH, helix–loop–helix domain, IG, immunoglobulin-like domain, *mant*, methylanthraniloyl, MD, molecular dynamics, NC, N-terminal cassette, NTR, N-terminal region, NTPase, nucleic-acid-dependent nucleotide triphosphatase, pre-mRNA, precursor messenger RNA, RNP, ribonucleoprotein complex, SEM, standard error of the mean, SF2, superfamily 2, sn, small nuclear, ss, single-stranded, T1, truncation 1, Tris, tris(hydroxymethyl)aminomethane, v/v, volume/volume, WH, winged-helix domain, wt, wild type

## Abstract

Brr2 is an essential Ski2-like RNA helicase that exhibits a unique structure among the spliceosomal helicases. Brr2 harbors a catalytically active N-terminal helicase cassette and a structurally similar but enzymatically inactive C-terminal helicase cassette connected by a linker region. Both cassettes contain a nucleotide-binding pocket, but it is unclear whether nucleotide binding in these two pockets is related. Here we use biophysical and computational methods to delineate the functional connectivity between the cassettes and determine whether occupancy of one nucleotide-binding site may influence nucleotide binding at the other cassette. Our results show that Brr2 exhibits high specificity for adenine nucleotides, with both cassettes binding ADP tighter than ATP. Adenine nucleotide affinity for the inactive C-terminal cassette is more than two orders of magnitude higher than that of the active N-terminal cassette, as determined by slow nucleotide release. Mutations at the intercassette surfaces and in the connecting linker diminish the affinity of adenine nucleotides for both cassettes. Moreover, we found that abrogation of nucleotide binding at the C-terminal cassette reduces nucleotide binding at the N-terminal cassette 70 Å away. Molecular dynamics simulations identified structural communication lines that likely mediate these long-range allosteric effects, predominantly across the intercassette interface. Together, our results reveal intricate networks of intramolecular interactions in the complex Brr2 RNA helicase, which fine-tune its nucleotide affinities and which could be exploited to regulate enzymatic activity during splicing.

RNA-dependent NTPases/helicases play a central role in all aspects of RNA metabolism (1). In many contexts they act as proofreaders, by timing structural transitions in RNAs or RNA–protein complexes (RNPs) or by sensing the authenticity of RNA/RNP networks (2). To these ends, their NTPase activities need to be finely tuned, which can involve intra- or intermolecular regulatory mechanisms (3). Mechanistic insights into nucleotide binding, hydrolysis, and product release are necessary to understand how NTPase rates can be regulated to meet cellular demands, but corresponding detailed studies are scarce. In particular, although allosteric regulation, *e.g.*, *via* accessory domains, is most likely a pervasive principle in RNA helicases, the underlying mechanistic details are often not known.

Ski2-like nucleic acid helicases constitute a family of superfamily 2 (SF2) helicases. All Ski2-like helicases display a common helicase core consisting of two RecA-like domains, a winged-helix (WH) domain and a helical bundle (HB or “ratchet”) domain arranged in a ring-like fashion (4). This domain arrangement has also been observed in structures of the DEAH/RHA family of SF2 helicases (5, 6, 7). Additionally, Ski2-like helicases can contain diverse accessory domains appended to or inserted into the conserved domains, which modulate or expand their molecular mechanisms or functions (3).

The two RecA-like domains contain 12 conserved sequence motifs (“helicase motifs”) involved in nucleotide tri-phosphate (NTP) and nucleic acid transactions (8). Nucleic acid substrates bind with 3′-to-5′ directionality across the first and second RecA-like domains and below the HB/ratchet domain, explaining the 3′-to-5′ unwinding directionality of these enzymes (9, 10, 11). Ski2-like helicases also contain a β-hairpin in the second RecA-like domain, which is shorter than a similar element in DEAH/RHA helicases and which is thought to act as a strand separation device that inserts between the two strands of a duplex when one strand of the substrate is pulled through the helicase unit (10, 12). Like DEAD-box helicases, Ski2-like helicases are thought to selectively utilize ATP due to the presence of a Q-motif (13).

Four Ski2-like RNA helicases have been identified in yeast: Ski2 (involved in RNA degradation and viral defense) (14), Slh1 (ribosome quality control) (15), Mtr4 (RNA degradation) (9), and Brr2 (pre-mRNA splicing) (16). In addition, an Mtr4 homolog, FRH, of *Neurospora crassa* is involved in the regulation of the circadian rhythm (17). Several additional Ski2-like enzymes are DNA helicases such as yeast Mer3/mammalian HFM1, involved in homologous recombination (18, 19), archaeal Hel308/eukaryotic POLQ-like helicase, involved in double-strand break repair (10, 20), and the mammalian ASCC3 helicase, associated with transcription regulation (21), DNA repair (22), ribosome quality control (23, 24, 25), nonfunctional ribosomal RNA decay (26), and viral defense (27). 3′-to-5′ translocation has been directly shown for Hel308 (10), Mer3/HFM1 (28), Mtr4 (29), and Brr2 (30, 31).

A common feature among Ski2-like RNA helicases is their integration into multiprotein assemblies. Brr2 is an integral component of the spliceosomal U5 snRNP (32, 33). Thus, during assembly of a spliceosome on a pre-mRNA substrate, Brr2 is recruited as part of the preassembled U4/U6•U5 tri-snRNP during formation of a precatalytic B complex spliceosome (34, 35, 36). Brr2 is essential for the conversion of the precatalytic to an activated spliceosome (37, 38), during which it unwinds the extensively base-paired U4/U6 di-snRNA (16, 39, 40). To this end, the enzyme binds to a single-stranded (ss) region of U4 snRNA and translocates in 3′-to-5′ direction (30, 40). Unlike other spliceosomal helicases, Brr2 remains stably associated with the spliceosome after incorporation of the tri-snRNP throughout the remaining stages of a splicing event (34, 36, 41, 42, 43, 44, 45, 46) and is required again during the two catalytic steps of splicing (47, 48) and during spliceosome disassembly (49). However, the Brr2 helicase activity *per se* may not be necessary during these later stages of splicing (45, 47). Thus, Brr2 has to be strictly regulated to prevent premature unwinding of U4/U6 in the tri-snRNP and to allow its repeated on- and off-switching during splicing.

Brr2 is a particularly large spliceosomal RNA helicase (ca. 245 kDa for the human enzyme). The helicase core of Brr2 is expanded by additional helix–loop–helix (HLH) and immunoglobulin-like (IG) domains, which form a Sec63-homology unit together with the HB/ratchet domain (50, 51). In addition, Brr2 contains two copies of the RecA1/2-WH-Sec63 units (cassettes) arranged in tandem (Fig. 1, *A* and *B*) (52). This dual-cassette organization is shared by only few other Ski2-like enzymes, including the RNA helicase Slh1 (53) and the DNA helicase ASCC3 (22). Only the N-terminal cassette (NC) of Brr2 is an active ATPase and RNA helicase (52), and the enzymatic activity of the NC alone is required for splicing *in vivo* (54). Nevertheless, the C-terminal cassette (CC) is essential for yeast viability (51), represents a versatile protein–protein interaction platform (55, 56), retains nucleotide-binding capacity (52, 57), and regulates the activity of the NC (52, 58). Therefore, the CC may be considered an intramolecular helicase cofactor in Brr2.

**Figure 1:**
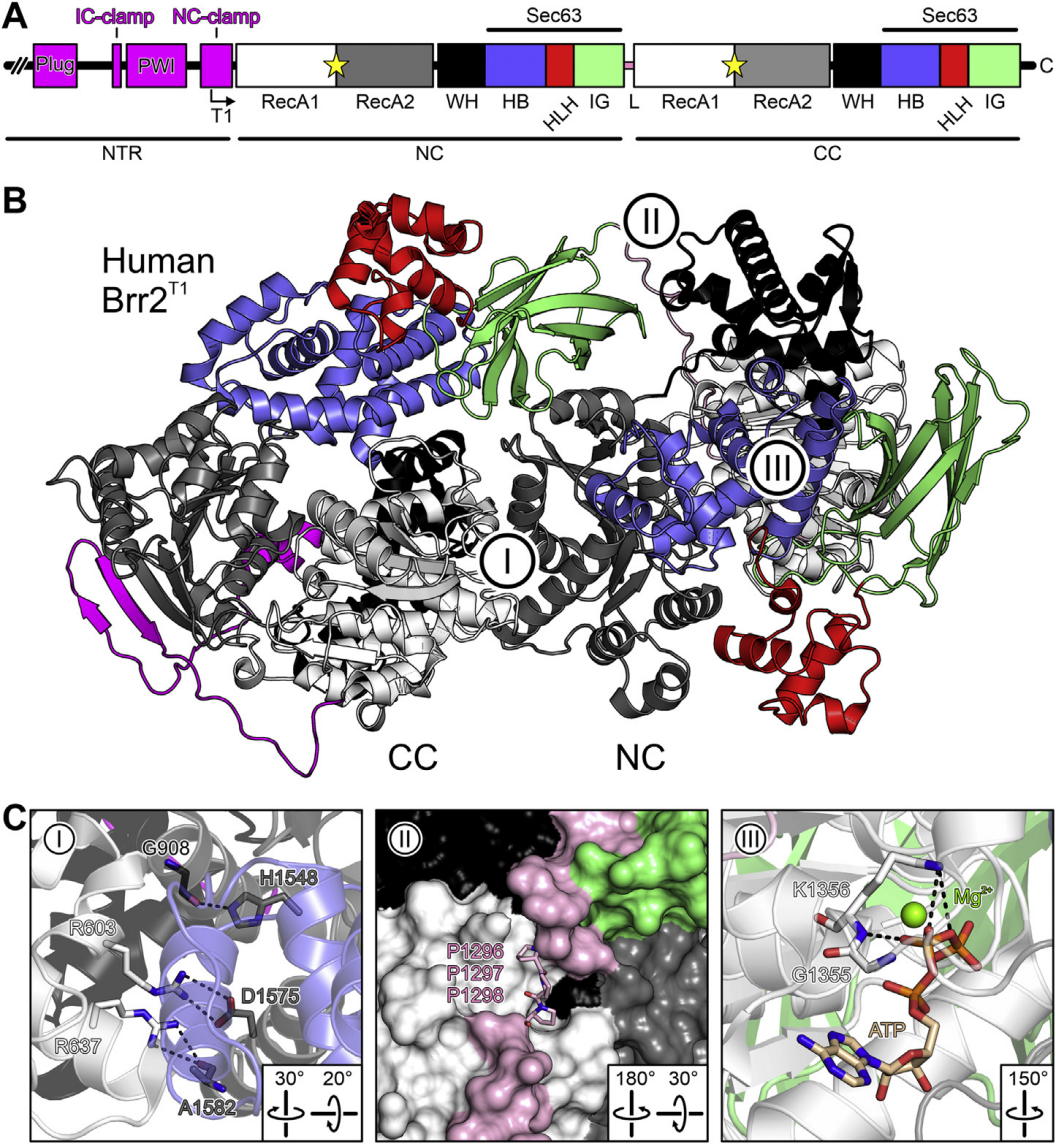
**Brr2 organization.***A*, domain organization of hBrr2. *Angled arrow*, starting position of the T1 truncation variant. *Yellow stars*, nucleotide-binding pockets. *B*, Structure of hBrr2^T1^ (PDB ID 4F91). I–III, regions mutated (see *C*). Domain coloring as in *A*. *C*, details of the mutated regions (I–III as in *A*, left). ATP-bound structure according to PDB ID 4F93 (region III, right). Relevant residues are shown as sticks. Domain coloring as in *A*. Residue coloring: Protein carbon, as the respective protein region; ATP carbon, *beige*; nitrogen, *blue*; oxygen, *red*; phosphorus, *orange*; magnesium ion, *green*. *Dashed lines*, hydrogen bonds or salt bridges. CC, C-terminal cassette; HB, helical bundle domain; HLH, helix–loop–helix domain; IG, immunoglobulin-like domain; L, linker; NC, N-terminal cassette; NTR, N-terminal region; RecA, RecA-like domain; WH, winged-helix domain.

Preceding its two helicase cassettes, Brr2 contains an evolutionarily conserved N-terminal region (NTR) of approximately 400 residues (Fig. 1*A*), capable of auto-inhibiting Brr2 (59). The NTR contains two folded domains connected by flexible regions and can fold back along one flank of the tandem helicase cassettes, blocking access of the RNA substrate and restricting conformational rearrangements required for RNA duplex unwinding (59). *In vivo*, the NTR is required for yeast viability, efficient splicing, Brr2 association with the U4/U6•U5 tri-snRNP, tri-snRNP stability (59, 60), retention of U5 and U6 snRNAs during spliceosome activation (38), and as a protein–protein interaction platform (61).

In spite of detailed structural analyses of the Brr2 helicase (52, 57, 59, 62), there is limited information about the molecular mechanisms underlying its NTPase cycle. Here, we used biophysical and computational methods to systematically characterize nucleotide binding to wild-type (wt) Brr2 and selected variants. Our results show that the Brr2 helicase cassettes have drastically different affinities to nucleotides and that ADP is preferred over ATP in both nucleotide-binding pockets. Residue exchanges at the intercassette interface reduced nucleotide association for both cassettes and mutations in the CC nucleotide-binding pocket interfered with nucleotide binding at the NC. We delineated structural communication lines in the enzyme by molecular dynamics (MD) simulations, which may mediate these long-range effects. Our results show that accessory regions in an RNA helicase (CC in the case of Brr2) can regulate nucleotide affinity and specificity in an intricate manner. The mechanistic principles we find underlying allosteric NTPase/helicase regulation in Brr2 might also be at work in related enzymes.

## Results

### Nucleotide specificity of human Brr2

We used recombinant human Brr2 (hBrr2), which we consider a good representative of all Brr2 orthologs, as Brr2 is evolutionarily highly conserved. Apart from full-length hBrr2, we investigated an N-terminally truncated variant (T1) that lacks most of the auto-inhibitory N-terminal region as well as the isolated helicase cassettes (hBrr2^NC^, hBrr2^CC^; Fig. 1*A*).

**Figure 2:**
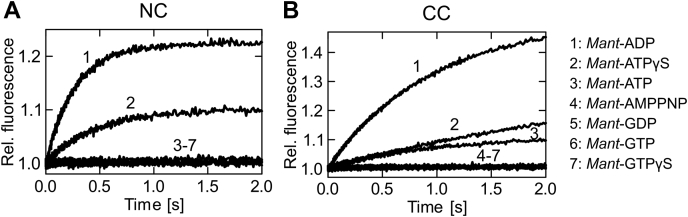
**Nucleotide specificity of the hBrr2 cassettes.***A* and *B*, time courses of *mant*-nucleotide binding to 0.5 μM nucleotide-free hBrr2^NC^ (*A*) and hBrr2^CC^ (*B*) measured by FRET from Trp to *mant*. 1, *mant*-ADP (5 μM); 2, *mant*-ATPγS (5 μM); 3, *mant*-ATP (5 μM); 4, *mant*-AMPPNP (5 μM); 5, *mant*-GDP (5 μM); 6, *mant*-GTP (5 μM); 7, *mant*-GTPγS (5 μM). The hBrr2 cassettes bind *mant*-ADP and *mant*-ATPγS but do not interact with *mant*-AMPPNP or *mant*-G nucleotides.

hBrr2 has two nucleotide-binding pockets, one in each of its cassettes (52) (Fig. 1*A*). The CC pocket is inactive in ATP hydrolysis (52). We first set out to directly test nucleotide-binding preferences for each cassette of hBrr2 in isolation, hBrr2^NC^ (residues 395–1324) and hBrr2^CC^ (residues 1282–2136), making use of fluorescence resonance energy transfer (FRET) from tryptophan residues in the vicinity of the NC and CC nucleotide-binding pockets (W817 in hBrr2^NC^ or W1652 and W1393 in hBrr2^CC^) to *mant*-nucleotides. In our experimental setups, excitation of tryptophans resulted in *mant* fluorescence increases upon binding of the labeled nucleotides; conversely, dissociation of the *mant*-nucleotides resulted in decreased FRET. Time courses of binding were recorded at a constant concentration of hBrr2^NC^ or hBrr2^CC^ (0.5 μM) with an excess of *mant*-nucleotide (5 μM; Fig. 2). Among the *mant*-nucleotides tested (ADP, ATP, ATPγS, AMPPNP, GDP, GTP, GTPγS), only *mant*-ADP and *mant*-ATPγS showed FRET with both hBrr2^NC^ and hBrr2^CC^ (Fig. 2, *A* and *B*). *mant*-ATP only yielded an FRET signal with hBrr2^CC^, but not with hBrr2^NC^ (Fig. 2, *A* and *B*). None of the guanosine nucleotides showed binding to either cassette.

These data show that both cassettes of hBrr2 possess a high specificity for adenine nucleotides. This finding is consistent with the presence of a so-called Q-motif in Ski2-like helicases, including in both helicase cassettes of hBrr2. The name-giving glutamine residue of the Q-motif forms hydrogen bonds to the N6 and N7 positions of the adenine base (63). Furthermore, the data suggest that both cassettes preferentially bind ADP over ATP.

### *Structures of* mant*-nucleotides bound at NC and CC*

Lack of an FRET signal from *mant*-AMPPNP with either cassette (Fig. 2, *A* and *B*) may indicate that AMPPNP is not a suitable ATP surrogate in the case of hBrr2. Similar observations have been made with GMPPNP and some GTPases (64). However, lack of an FRET signal from *mant*-ATP with hBrr2^NC^ was surprising, given that *mant*-ATPγS yielded a signal (Fig. 2, *A* and *B*). Based on this observation, we set out to investigate whether *mant*-nucleotides that give rise to FRET signals exhibit the same binding poses as the corresponding unlabeled nucleotides and whether the *mant* moieties influence nucleotide binding. To this end, we determined crystal structures of hBrr2 in complex with ADP, ATPγS, *mant*-ADP, and *mant*-ATPγS at 2.5–2.8 Å resolution (Table S1). For crystallographic analyses, we used N-terminally truncated hBrr2^T1^ in complex with the Jab1 domain of the hPrp8 protein lacking a Brr2-inhibiting C-terminal tail (hJab1^ΔC^). The hBrr2^T1^-hJab1^ΔC^ complex yields well-diffracting crystals under low-salt conditions (58), suitable for nucleotide binding.

**Figure 3:**
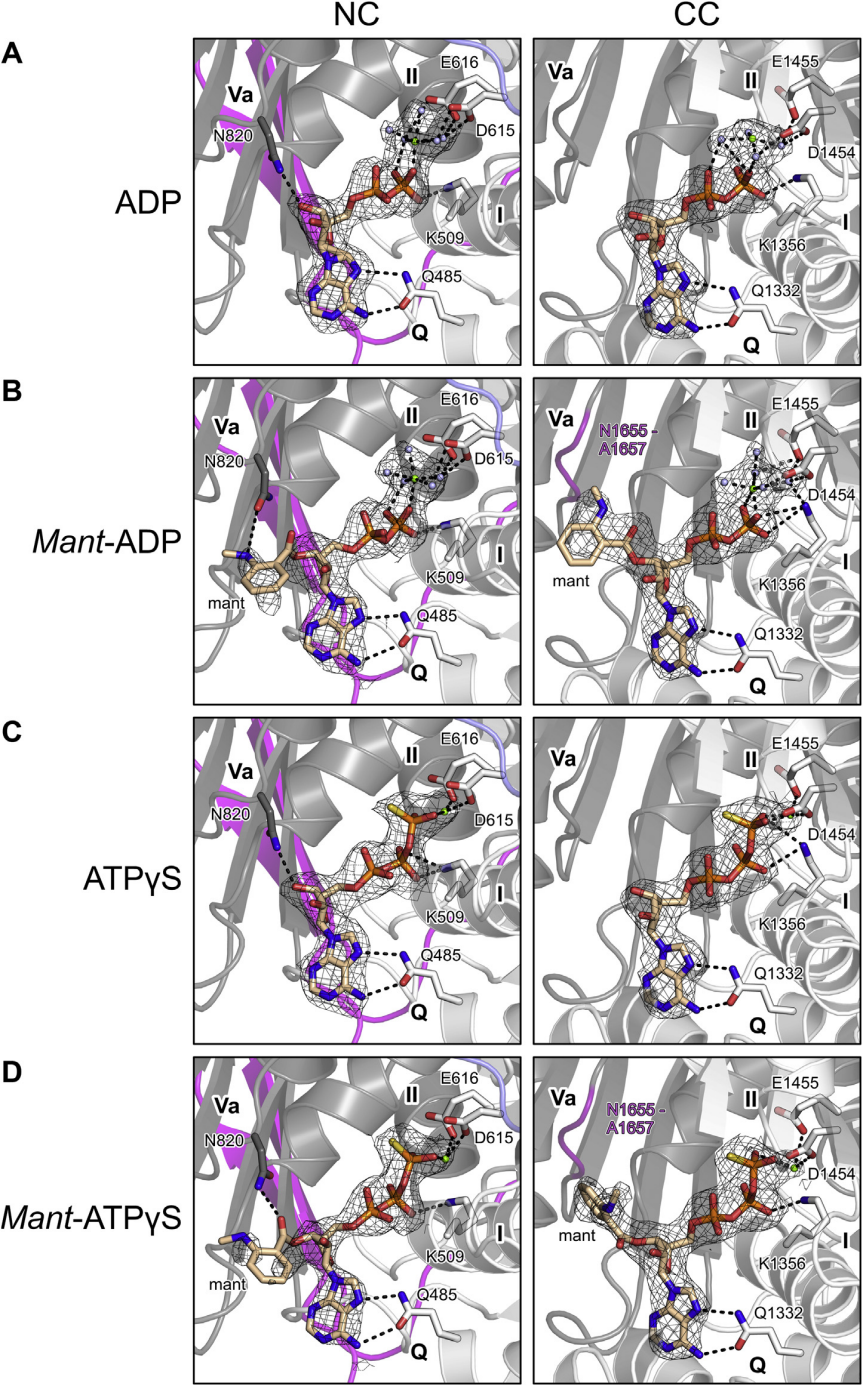
**Nucleotide-bound structures.***A–D*, binding poses of the indicated nucleotides at the N- and C-terminal cassettes of an hBrr2^T1^-hJab1^ΔC^ complex. Meshes, final 2mF_o_-DF_c_ electron density maps at the 1σ level covering the nucleotides. *Left panels*, nucleotides bound at NC; *right panels*, nucleotides bound at CC. Nucleotides, magnesium ions, selected waters, and selected Brr2 residues are shown as sticks or small spheres. Domain coloring as in Figure 1*A*. Atom/ion coloring: Protein carbon, as the respective protein region; nucleotide carbon, *beige*; nitrogen, *blue*; oxygen, *red*; phosphorus, *orange*; magnesium ion, *green*; water oxygen, *light blue*. *Dashed lines*, hydrogen bonds, salt bridges, or ion coordination.

Electron densities for the phosphate groups, the riboses, and the adenine bases were well defined in both NC and CC for all nucleotides analyzed (Fig. 3, *A*–*D*). At both cassettes, accommodation of the phosphate/ribose/base portions of *mant*-ADP and *mant*-ATPγS was essentially unaltered compared with ADP and ATPγS (Fig. 3, *A*–*D*; Fig. S1), showing that the *mant* units do not influence how the nucleotides are positioned in the binding pockets. Moreover, ATPγS and the ATPγS portion of *mant*-ATPγS are accommodated as expected for ATP (52). The structures also revealed that two tryptophan residues (W1393 and W1652) reside within 20 Å distance of the CC nucleotide-binding pocket, while only one tryptophan residue (W817) is close to the NC nucleotide-binding pocket (Fig. S2), providing an explanation for higher FRET at the CC.

Additionally, electron densities for the *mant* units of *mant*-ADP and *mant*-ATPγS were less defined in the NC compared with the CC (Fig. 3, *A*–*D*), indicating that the fluorophores remain more mobile when *mant*-nucleotides are accommodated at the NC compared with the CC. With *mant* nucleotides bound at the NC, N820 (helicase motif Va) fosters a hydrogen bond to the *mant* moieties instead of the sugar units of unlabeled nucleotides (Fig. 3, *A*–*D*, left). At the CC, *mant* moieties engage in additional van der Waals (vdW) contacts to the helicase motif Va residues (Fig. 3, *B* and *D*, right).

Based on these results, we conclude that the *mant* moieties do not significantly alter the positioning of ADP or ATPγS in the nucleotide-binding pockets, but may lead to higher affinities of *mant*-nucleotides compared with the corresponding unmodified nucleotides by fostering additional contacts. The better defined electron densities for the *mant* moieties in the CC compared with the NC suggest that the *mant* units have a relatively larger effect on nucleotide affinities to the CC compared with the NC. Furthermore, comparable distances (within ±0.2 Å) between *mant* moieties and close tryptophan residues in the structures with *mant*-ADP and *mant*-ATPγS (Fig. S2) suggest that higher FRET signals obtained with *mant*-ADP compared with *mant*-ATPγS (Fig. 2, *A* and *B*) are due to a lower occupancy of the nucleotide-binding pockets in the case of *mant*-ATPγS under the chosen conditions, as a consequence of a lower affinity of ATPγS compared with ADP. In addition, we suggest that hBrr2^NC^ exhibits higher conformational flexibility than the NC in context of a dual-cassette Brr2 construct (hBrr2^T1^ or hBrr2^FL^) and that, therefore, *mant*-ATP is not sufficiently stably bound at the isolated NC to yield an FRET signal (Fig. 2*A*). We were predominantly interested in affinity changes due to allosteric effects, which we expect to affect *mant*-nucleotides and unmodified nucleotides in a similar manner. We, thus, concluded that *mant*-ADP and *mant*-ATPγS are suitable surrogates for studying the binding of ADP and ATP to the Brr2 nucleotide-binding pockets and, therefore, employed these nucleotides in subsequent experiments.

### Affinities of the isolated N- and C-terminal cassettes of hBrr2 for adenine nucleotides

Our initial investigations on the nucleotide specificity of the hBrr2 cassettes in isolation indicated that hBrr2^NC^ and hBrr2^CC^ bind nucleotides with different rates. To determine the nucleotide association rate constants, *k*_1_^*NC*^ and *k*_1_^*CC*^, and the equilibrium affinity constants, we investigated *mant*-ATPγS/*mant*-ADP binding to hBrr2^NC^ and hBrr2^CC^ using strategies previously described for G nucleotide binding to translation factor GTPases (65, 66, 67, 68).

**Figure 4:**
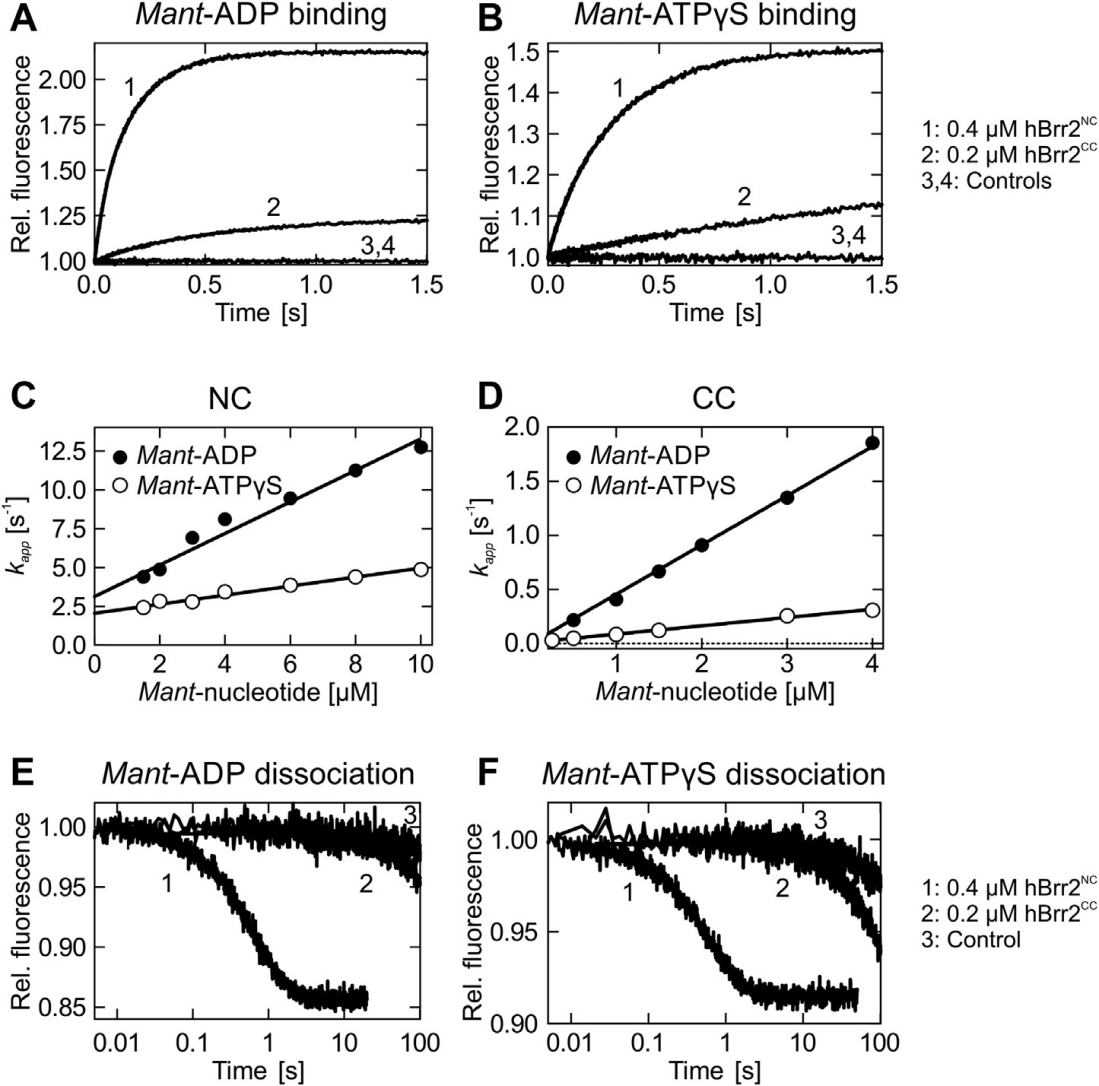
**Kinetics of *mant*-ADP and *mant*-ATPγS interaction with hBrr2**^**NC**^**and hBrr2**^**CC**^**.***A* and *B*, time courses of 5 μM *mant*-ADP (*A*) or *man*t-ATPγS (*B*) binding to hBrr2^NC^ (1; 0.4 μM) and hBrr2^CC^ (2; 0.2 μM) measured by FRET from Trp to *mant*. Controls correspond to binding experiments with unlabeled ADP or ATPγS (3 and 4). *C* and *D*, individual nucleotide binding traces were fitted to single exponentials and the dependencies of the apparent rate constants on nucleotide concentration for hBrr2^NC^ (*C*) and hBrr2^CC^ (*D*) were fitted by a linear equation, *k*_*app*_ = *k*_1_[mant-nucleotide]+*k*_−1_, in which *k*_1_ is derived from the slope and *k*_−1_ from the *y*-axis intercept. *Closed circles*, *mant*-ADP; *open circles*, *mant*-ATPγS. Values represent means ± SD of at least three independent measurements. Error bars that are not visible are smaller than the symbols. *E* and *F*, dissociation of 5 μM *mant*-ADP (*E*) or *man*t-ATPγS (*F*) from hBrr2^NC^ (1; 0.4 μM hBrr2^NC^) and hBrr2^CC^ (2; 0.2 μM) in the presence of the respective unlabeled nucleotide in excess (100 μM). Control experiments (3) were carried out in the absence of unlabeled nucleotide (curves shown are for hBrr2^CC^).

We acquired time courses of binding at a constant concentration of nucleotide-free hBrr2^NC^ or hBrr2^CC^ and varying concentrations of *mant*-ATPγS/*mant*-ADP under pseudo-first-order conditions (Fig. 4, *A* and *B*). The time dependencies of FRET were analyzed by exponential fitting to calculate the apparent rate constant of nucleotide binding to either cassette at each nucleotide concentration (*k*_*app*_^*NC*^ and *k*_*app*_^*CC*^). hBrr2^NC^ and hBrr2^CC^ time traces were best fit with a single-exponential equation, consistent with a one-step binding model (Fig. 4, *A* and *B*). The bimolecular association rate constants, *k*_1_^*NC*^ and *k*_1_^*CC*^, were determined from the slopes of the linear dependences of *k*_*app*_^*NC*^ and *k*_*app*_^*CC*^, respectively, on the concentration of *mant*-ATPγS/*mant*-ADP (Fig. 4, *C* and *D*; Table 1). The values obtained indicate that both isolated hBrr2^NC^ and hBrr2^CC^ bind ADP faster than ATPγS. Furthermore, hBrr2^NC^ binds both tested nucleotides faster than hBrr2^CC^.

**Table 1:** Kinetics of nucleotide binding and release

Protein	Region affected	Data fitting^a^	*mant*-ADP				*mant*-ATPγS			
			NC		CC		NC		CC	
			*k*_1_ [μM^−1^ s^−1^]	*k*_−1_ [s^−1^]	*k*_1_ [μM^−1^ s^−1^]	*k*_−1_ 10^-3^ [s^−1^]	*k*_1_ [μM^−1^ s^−1^]	*k*_−1_ [s^−1^]	*k*_1_ [μM^−1^ s^−1^]	*k*_−1_ 10^−3^ [s^−1^]
hBrr2^NC^	-	SE	1.0 ± 0.1	1.9 ± 0.1	-	-	0.3 ± 0.02	1.6 ± 0.02	-	-
hBrr2^CC^	-	SE	-	-	0.5 ± 0.01	2.0 ± 0.2	-	-	0.1 ± 0.001	2.0 ± 0.2
hBrr2^FL^	-	DE	2.8 ± 0.03	1.5 ± 0.02	0.4 ± 0.01	1.0 ± 0.1	1.0 ± 0.02	1.5 ± 0.04	0.1 ± 0.01	1.0 ± 0.1
hBrr2^T1^	-	DE	2.5 ± 0.1	1.3 ± 0.02	0.4 ± 0.02	1.0 ± 0.1	2.5 ± 0.03	1.2 ± 0.04	0.1 ± 0.01	0.9 ± 0.01
R603A	Cassette interface	DE	0.2 ± 0.03	1.4 ± 0.04	0.02 ± 0.001	1.0 ± 0.1	0.3 ± 0.03	1.0 ± 0.04	0.02 ± 0.001	1.0 ± 0.2
R637A	Cassette interface	DE	3.4 ± 0.2	1.2 ± 0.03	0.5 ± 0.01	1.0 ± 0.1	0.4 ± 0.04	1.0 ± 0.1	0.02 ± 0.001	1.0 ± 0.04
S1087L^b^	NC HB domain	DE	2.7 ± 0.1	1.4 ± 0.04	0.5 ± 0.01	1.0 ± 0.03	2.2 ± 0.21	1.4 ± 0.04	0.1 ± 0.004	1.0 ± 0.4
PPP1296-8AAA	Inter-cassette linker	DE	2.9 ± 0.1	2.2 ± 0.1	0.52 ± 0.003	2.0 ± 0.04	0.5 ± 0.03	2.2 ± 0.1	0.03 ± 0.002	2.0 ± 0.4
GK1355-6QE	CC ATP pocket	SE	1.3 ± 0.1	1.5 ± 0.02	-	-	0.4 ± 0.02	1.7 ± 0.1	-	-
H1548A	Cassette interface	DE	3.3 ± 0.3	1.2 ± 0.1	0.5 ± 0.01	1.0 ± 0.3	0.2 ± 0.02	1.3 ± 0.1	0.02 ± 0.001	1.0 ± 0.03

a SE, single-exponential; DE, double exponential.

b S1087L in the N-terminal HB domain originates from a retinitis pigmentosa-linked mutation of hBrr2 and was carried along as a negative control.

The dissociation rate constants were determined by preforming hBrr2^NC/CC^ complexes with *mant*-ADP or *mant*-ATPγS and mixing the complexes with an excess of the respective unlabeled nucleotide, using a stopped-flow apparatus. Under these conditions, the rate of ADP/ATPγS binding is limited by the rate of *mant*-ADP/*mant*-ATPγS dissociation from hBrr2^NC^ or hBrr2^CC^. Rebinding of the *mant*-nucleotide is negligible due to the large excess of unlabeled nucleotide. Thus, the rate by which the *mant* fluorescence decreases equals the dissociation rate of the *mant*-nucleotide. Dissociation rate constants were obtained by single-exponential fitting of the time courses (Fig. 4, *E* and *F*; Table 1). hBrr2^NC^ dissociation rates (*k*-_1_^*NC*^) were 1.9 ± 0.1 s^−1^ and 1.6 ± 0.02 s^−1^ for *mant*-ADP and *mant*-ATPγS, respectively. ADP and ATPγS dissociation rates for hBrr2^CC^ (*k*-_1_^*CC*^) were three orders of magnitude lower than for hBrr2^NC^, 2 ± 0.2 10^−3^ s^−1^ (Fig. 4, *E* and *F*; Table 1). This observation is explained by comparing the NC and CC nucleotide-binding pockets (Fig. S3, *A* and *B*), while N1655 (motif Va) of the CC is remote from the nucleotide as opposed to the equivalent N820 of the NC, residues P1694/L1695 (following motif VI) of the CC approach and contact the adenine base, unlike the equivalent P859/Q860 of the NC. P1694/L1695 thus appear to lock the nucleotide in the CC-binding pocket.

The *K*_*d*_ values for the interaction of ADP and ATPγS with hBrr2^NC^ and hBrr2^CC^ were calculated from the ratios of dissociation and association rate constants (*K*_*d*_^NC^ = *k*-_1_^*NC*^/*k*_1_^*NC*^; *K*_*d*_^CC^ = *k*-_1_^*CC*^/*k*_1_^*CC*^). Both hBrr2^NC^ (3-fold) and hBrr2^CC^ (7.5-fold) displayed a higher affinity toward ADP compared with ATPγS (Table 2). Furthermore, hBrr2^CC^ has about 500-fold and about 200-fold higher affinities for ADP and ATPγS, respectively, as compared with hBrr2^NC^, largely determined by the dissociation rates (*k*-_1_^*CC*^) for *mant*-nucleotides (Table 2). Thus, although the two helicase cassettes are structurally similar (Fig. S3, *A* and *B*), they differ over more than two orders of magnitude in their capacity to interact with adenine nucleotides.

**Table 2:** Affinities of hBrr2 variants for *mant*-ADP and *mant*-ATPγS

Protein	*mant*-ADP		*mant*-ATPγS	
	NC	CC	NC	CC
	*K*_*d*_ [μM]	*K*_*d*_ [nM]	*K*_*d*_ [μM]	*K*_*d*_ [nM]
hBrr2^NC^	2.0 ± 0.2	-	5.8 ± 0.4	-
hBrr2^CC^	-	4 ± 0.4	-	30 ± 3
hBrr2^FL^	0.5 ± 0.01	2 ± 0.2	1.6 ± 0.1	10 ± 1
hBrr2^T1^	0.5 ± 0.02	2 ± 0.3	0.5 ± 0.02	10 ± 1
R603A	6.3 ± 0.8	50 ± 4	4.1 ± 0.5	50 ± 10
R637A	0.4 ± 0.2	2 ± 0.1	2.6 ± 0.03	40 ± 2
S1087L^a^	0.5 ± 0.02	2 ± 0.1	0.6 ± 0.1	10 ± 4
PPP1296-8AAA	0.8 ± 0.05	4 ± 0.1	4.3 ± 0.4	60 ± 10
GK1355-6QE	1.2 ± 0.05	-	4.4 ± 0.3	-
H1548A	0.4 ± 0.04	2 ± 0.6	5.9 ± 0.6	40 ± 2

a S1087L in the N-terminal HB domain originates from a retinitis pigmentosa-linked mutation of hBrr2 and was carried along as a negative control.

### Interaction of mant-ATPγS/mant-ADP with hBrr2^FL^ and hBrr2^T1^

**Figure 5:**
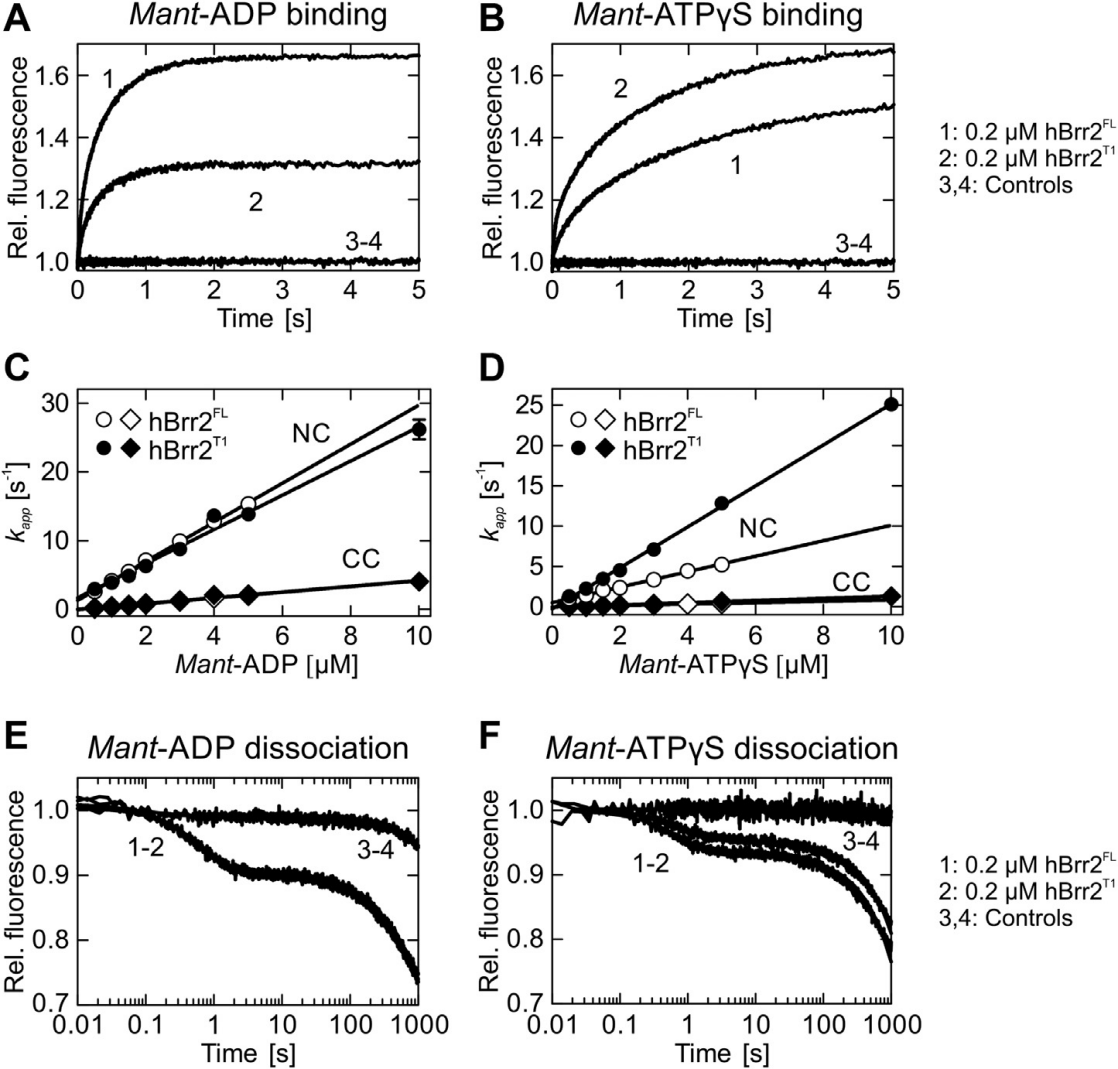
**Kinetics of *mant*-ADP and *mant*-ATPγS interaction with hBrr2**^**FL**^**and hBrr2**^**T1**^**.***A* and *B*, time courses of 5 μM *mant*-ADP (*A*) or *man*t-ATPγS (*B*) binding to hBrr2^FL^ (1; 0.2 μM) and hBrr2^T1^ (2; 0.2 μM) measured by FRET from Trp to *mant*. Controls were performed with unlabeled ADP or ATPγS (3, 4). *C* and *D*, individual nucleotide binding traces were fitted to double exponential equations, and the dependence of the apparent rate constants of the NC (*circles*) and CC (*diamonds*) on nucleotide concentration were fitted by a linear equation. *Open symbols*, hBrr2^FL^; *closed symbols*, hBrr2^T1^. The cassettes of hBrr2^FL^ and hBrr2^T1^ bind nucleotides with different velocities as observed for the variants containing either one of the cassettes, hBrr2^NC^ and hBrr2^CC^. While the CC binds *mant*-ADP and *man*t-ATPγS with similar rates, the NC in hBrr2^T1^ binds *mant*-ATPγS faster than the NC in hBrr2^FL^. Values represent means ± SD of at least three independent measurements. Error bars that are not visible are smaller than the symbols. *E* and *F*, dissociation of 5 μM *mant*-ADP (*E*) or *mant*-ATPγS (*F*) from hBrr2^FL^ (1; 0.2 μM) and hBrr2^T1^ (2; 0.2 μM) in the presence of the respective unlabeled nucleotide in excess (100 μM). Control experiments (3 and 4) were carried out in the absence of unlabeled nucleotide.

We next studied nucleotide-binding behavior to hBrr2^FL^ and hBrr2^T1^, using the same detection principles as described above. hBrr2^FL^ comprises the full-length helicase, whereas hBrr2^T1^ lacks the NTR except for a segment that meanders along the NC (NC-clamp; Fig. 1*A*). The time courses of nucleotide binding were obtained at a constant concentration of nucleotide-free hBrr2^FL^ or hBrr2^T1^ and a varying concentration of *mant*-ADP/*mant*-ATPγS. For both hBrr2^FL^ and hBrr2^T1^, biphasic time dependencies were observed (Fig. 5, *A* and *B*), consistent with the nucleotides binding to the NC and CC of hBrr2. Nonlinear regression analysis using two exponential terms yielded two apparent rate constants, *k*_*app*1_ and *k*_*app*2_, for each time trace. To assign each *k*_*app*_ to NC or CC, we integrated the results as for the isolated cassette constructs. *k*_*app*1_ dependencies over nucleotide concentration were in the same range as those obtained with the isolated hBrr2^NC^, whereas *k*_*app*2_ dependencies roughly agreed with those of hBrr2^CC^, allowing assignment of each apparent rate constant to adenine nucleotide interaction with each cassette. The bimolecular association rate constants, *k*_1_^*NC*^ and *k*_1_^*CC*^, were determined from the linear concentration dependences of *k*_*app*_^*NC*^ and *k*_*app*_^*CC*^ on the concentration of *mant*-nucleotides (Fig. 5, *C* and *D*; Table 1).

The slope of the linear fitting of *k*_*app*_^*NC*^, corresponding to the binding of *mant*-ADP to the NC in the context of hBrr2^FL^ and hBrr2^T1^, indicated association rate constants *k*_1_^*NC*^ of 2.8 ± 0.03 μM^−1^ s^−1^ and 2.5 ± 0.11 μM^−1^ s^−1^, respectively. The *k*_1_^*NC*^ values obtained for *mant*-ATPγS binding to the NC of hBrr2^FL^ and hBrr2^T1^ were 1.0 ± 0.02 μM^−1^ s^−1^ and 2.5 ± 0.03 μM^−1^ s^−1^, respectively. The *mant*-ADP and *mant*-ATPγS association rates to the NC in the context of hBrr2^FL^ and hBrr2^T1^ are thus 8- to 3-fold higher than the rates of nucleotide binding to isolated hBrr2^NC^ (*k*_1_^*NC*^ of 1.0 ± 0.07 μM^−1^ s^−1^ and 0.3 ± 0.02 μM^−1^ s^−1^, respectively, for *mant*-ADP and *mant*-ATPγS), suggesting that the CC slightly improves the ability of the NC to bind nucleotides. These observations are in line with previous findings that hBrr2^NC^ exhibits lower intrinsic and stimulated ATPase activities and lower U4/U6 di-snRNA unwinding activity compared with hBrr2^T1^ (52).

*Mant*-ADP binding to the CC of hBrr2^FL^ and hBrr2^T1^ was faster compared with *mant*-ATPγS, with an association rate constant, *k*_1_^*CC*^, of 0.4 ± 0.01 μM^−1^ s^−1^ for both hBrr2 variants. The hBrr2^FL^ and hBrr2^T1^ rate constants for *mant*-ATPγS binding to the CC, *k*_1_^*CC*^, were similar (0.1 ± 0.01 μM^−1^ s^−1^). These values agree well with the *k*_1_^*CC*^ obtained for *mant*-ADP and *mant*-ATPγS binding to the isolated hBrr2^CC^, 0.5 ± 0.01 μM^−1^ s^−1^ and 0.1 ± 0.001 μM^−1^ s^−1^, respectively, indicating that presence of the NC does not significantly influence nucleotide binding at the CC.

Nucleotide dissociation from hBrr2^FL^ and hBrr2^T1^ was studied as before with the isolated hBrr2^NC^ and hBrr2^CC^ constructs. Nucleotide dissociation rate constants, *k*-_1_^*NC*^ and *k*-_1_^*CC*^, were determined upon mixing hBrr2^FL/T1^-*mant*-ADP or hBrr2^FL/T1^-*mant*-ATPγS with an excess of the respective unlabeled nucleotide. The release of the labeled nucleotide from both hBrr2 nucleotide-binding pockets resulted in a two-phase fluorescence decrease, consistent with the dissociation of *mant*-ADP or *mant*-ATPγS from the two hBrr2 cassettes (Fig. 5, *E* and *F*). As nucleotide dissociation from the isolated NC was much faster than from the isolated CC (see above), the dissociation rate constants of the fast phases were assigned to the NC, *k*-_1_^*NC*^. The nucleotide dissociation rate constants *k*-_1_^*NC*^ for hBrr2^FL^ and hBrr2^T1^ were very similar, around 1.5 ± 0.02 s^−1^ (hBrr2^FL^) and 1.3 ± 0.02 s^−1^ (hBrr2^T1^) for *mant*-ADP and around 1.5 ± 0.04 s^−1^ (hBrr2^FL^) and 1.2 ± 0.04 s^−1^ (hBrr2^T1^) for *mant*-ATPγS (Table 1). These values are in agreement with the *k*-_1_^*NC*^ obtained for *mant*-ADP and *mant*-ATPγS in the isolated hBrr2^NC^ (1.9 ± 0.1 s^−1^ and 1.6 ± 0.02 s^−1^, respectively). The second phase indicated very slow nucleotide release from the CC in the context of hBrr2^FL^ and hBrr2^T1^, with a dissociation rate constant, *k*-_1_^*CC*^, of about 1 ± 0.1 10^−3^ s^−1^ for ADP and ATPγS, similar to the values associated with the very slow release of nucleotides from isolated hBrr2^CC^. Therefore, the CC appears to be primarily a binding site where nucleotides interact and remain bound due to the low dissociation rate constants. The NC is a site of high turnover with rapid nucleotide binding and rapid release, consistent with its abilities to hydrolyze ATP and promote RNA unwinding (52).

The *K*_*d*_ values for the interaction of ADP and ATPγS with hBrr2^FL^ and hBrr2^T1^ were also calculated from the ratios of the association (*k*_1_^*NC*^ and *k*_1_^*CC*^) and dissociation rate constants (*k*-_1_^*NC*^ and *k*-_1_^*CC*^). Due to the small dissociation rate constants, *k*-_1_^*CC*^, the CC nucleotide-binding pockets in hBrr2^FL^/hBrr2^T1^ have about 270-fold/260-fold higher affinities for ADP and about 160-fold/50-fold higher affinities for ATPγS, respectively, compared with the NC pocket (Table 2).

Both nucleotide-binding pockets of hBrr2^FL^ exhibit slightly higher affinities for ADP than ATPγS, with a 3-fold and 5-fold preference for ADP in the NC and CC, respectively. Upon NTR truncation (hBrr2^T1^), ADP and ATPγS bind with equal affinities to the NC, while the CC still has a 5-fold higher affinity for ADP. Thus, presence of the NTR seems to bias the relative NC nucleotide preference toward ADP, which may contribute to its function as an auto-inhibitory device.

### Residues in the intercassette interface and linker modulate nucleotide binding at both cassettes

Direct contacts of the NC RecA1 and WH domains to the CC RecA2 domain observed in Brr2 crystal structures (52, 57, 59, 62) are candidate sites for intercassette communication (Fig. 1*C*, left). Indeed, single alanine substitutions expected to weaken interactions between the NC RecA1 and the CC RecA2 domains have been reported to reduce the helicase activity of hBrr2^T1^ without affecting its RNA-binding properties (52). To test the effects of intercassette contacts on nucleotide binding, we mutated R603 (RecA1^NC^; contacts D1575 of RecA2^CC^), R637 (RecA1^NC^; contacts A1582 of RecA2^CC^), and H1548 (RecA2^CC^; contacts G908 of WH^NC^) individually to alanines (Fig. 1*C*, left).

The linker between NC and CC also establishes interaction networks between the cassettes, and alterations to the linker can likewise affect hBrr2^T1^ helicase activity both positively and negatively (52). To test the role of the linker in nucleotide binding, we exchanged PPP1296-8 (upper part of the linker running between IG^NC^ and RecA1^CC^) to alanines (Fig. 1*C*, middle). hBrr2^T1^ mutants were expressed, purified, and exhibited cooperative transitions with comparable melting temperatures in thermofluor-based thermal melting experiments (Fig. S4). Furthermore, equilibrium CD spectra were indicative of a high content of regular secondary structure in all hBrr2 variants. These data indicate that all hBrr2^T1^ variants tested herein were well folded and that mutant effects were not simply a result of a loss of stable tertiary structure.

**Figure 6:**
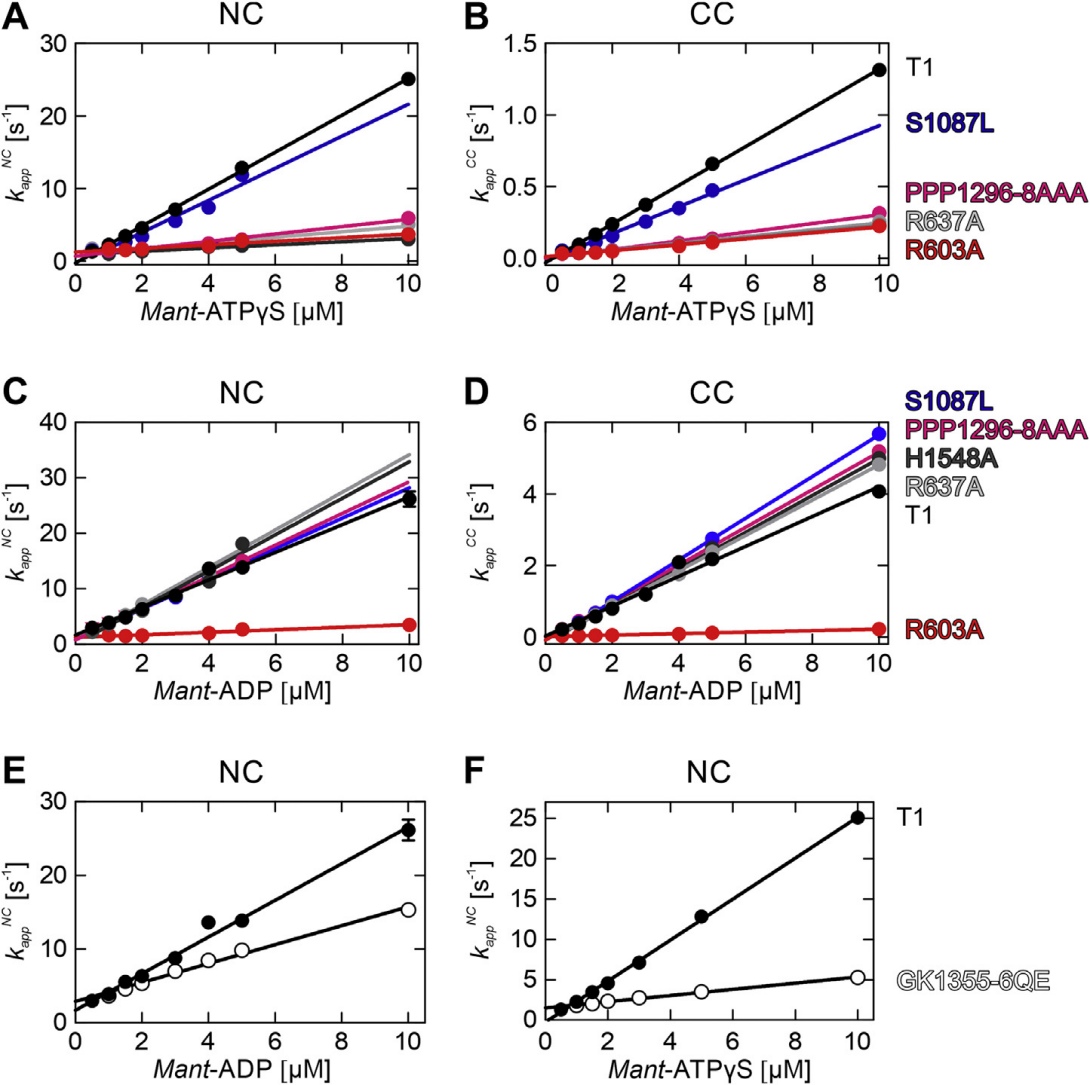
**Effects of hBrr2**^**T1**^**mutations on the kinetics of *mant*-ADP and *mant*-ATPγS binding.***A* and *B*, apparent rate constants (*k*_*app*_^*NC*^ and *k*_*app*_^*CC*^) of *mant*-ATPγS binding to the respective cassettes in hBrr2^T1^ and variants thereof. Binding of *mant*-ATPγS to either NC or CC is affected by all intercassette mutations tested. S1087L in the N-terminal HB domain originates from a retinitis pigmentosa-linked mutation of hBrr2 and was carried along as a negative control. *C* and *D*, apparent rate constants (*k*_*app*_^*NC*^ and *k*_*app*_^*CC*^) of *mant*-ADP binding to the respective cassettes of hBrr2^T1^ and variants thereof. While most intercassette mutants bind *mant*-ADP at similar rates as hBrr2^T1^, *mant*-ADP binding is almost completely abrogated at both NC and CC in R603A (*red*). hBrr2^T1^, *black*; S1087L (control with residue exchange in the N-terminal HB ratchet helix), *blue*; R603A (cassette interface NC), *red*; R637A (cassette interface NC), *light gray*; PPP1296-8AAA (linker), *magenta*; H1548A (cassette interface CC), *dark gray*. Coloring as in *A* and *B*, S1087L in the N-terminal HB domain originates from a retinitis pigmentosa-linked mutation of hBrr2 and was carried along as a negative control. *E* and *F*, apparent rate constants (*k*_*app*_^*NC*^) of *mant*-ADP and *mant*-ATPγS binding to the NC of hBrr2^T1^ (*closed circles*) and its GK1355-6QE variant (altered CC nucleotide binding pocket; *open circles*). The time courses of nucleotide binding to GK1355-6QE were fitted by a single exponential indicating no detectable nucleotide binding at the CC, as expected due to the two residue exchanges in the CC-binding pocket. As GK1355-6QE only has an intact NC nucleotide-binding pocket, only the hBrr2^T1^*k*_*app*_^*NC*^ is shown for comparison. GK1355-6QE shows reduced rates for nucleotide binding at the NC, suggesting long-range modulation of NC nucleotide binding by nucleotide binding at the CC in hBrr2^T1^. Values represent means ± SD of at least three independent measurements. Error bars that are not visible are smaller than the symbols.

Rate constants of nucleotide binding and dissociation to hBrr2^T1^ mutants were determined as described above for the wt hBrr2^T1^ protein. All association and dissociation time courses recorded for interface and linker mutants were best described by double-exponential fitting, representing the nucleotide binding and dissociation events at the NC and CC. The bimolecular association rate constants for nucleotide binding at the NC and CC, *k*_1_^*NC*^ and *k*_1_^*CC*^, were determined from the slope of the linear concentration dependence of the *k*_*app*_^*NC*^ and *k*_*app*_^*CC*^ (Table 1). Strikingly, all variants, R603A (NC-RecA1), R637A (NC-RecA1), PPP1296-1298AAA (linker), and H1548A (CC-RecA2; Fig. 1*C*, left and middle), exhibited reduced ATPγS association rate constants to the NC and CC, while the dissociation rate constants for both cassettes were similar to those of wt hBrr2^T1^ (Fig. 6, *A* and *B*). As a consequence, the resultant *K*_*d*_'s showed that the variant proteins bind ATPγS with lower affinity compared with wt hBrr2^T1^. Interestingly, only one mutant had an effect on ADP binding. The R603A variant (Fig. S3*C*) displayed about 10-fold and about 20-fold lower ADP association rate constants for NC and CC, respectively, compared with wt hBrr2^T1^ (Fig. 6, *C* and *D*; Table 1). ADP dissociation rate constants of both cassettes were largely unaffected by the mutations (Fig. 6, *C* and *D*; Table 1). In summary, all mutants reduced binding of ATPγS to either cassette, one mutant (R603A) also reduced ADP binding to both cassettes, while the release of ADP or ATPγS from either cassette was largely unaffected. Thus, intercassette contacts appear to configure the adenine nucleotide-binding pockets for ATP binding. Interestingly, ATPγS association rate constants to hBrr2^T1^ variants with diminished intercassette contacts resemble those of the isolated hBrr2^NC^, suggesting that the regulatory CC requires those intercassette contacts to modulate the NC.

### Mutation of the C-terminal nucleotide-binding pocket affects nucleotide binding at the N-terminal cassette

Mutations in motif I (Walker A motif or P-loop) of NTP-binding proteins and NTPases are known to interfere with nucleotide binding. In particular, a lysine residue in this motif is crucial for phosphate coordination and nucleotide stabilization (69). To test if nucleotide occupancy at the CC affects nucleotide binding at the NC, we generated a GK1355-6QE variant of hBrr2^T1^, bearing residue exchanges in motif I of the CC expected to abrogate nucleotide binding at the CC (Fig. 1*C*, right). Consistently, hBrr2^T1^ GK1355-6QE nucleotide association and dissociation time courses were best described by a single exponential, indicating nucleotide binding at the NC only. The association rate constants for *mant*-ADP and *mant*-ATPγS derived from the linear *k*_*app*_^*NC*^ concentration dependence were more than 6-fold and about 2-fold slower, respectively, compared with the rates seen with wt hBrr2^T1^ (Fig. 6, *E* and *F*; Table 1). The dissociation rate constants for ATPγS and ADP remained unchanged, as observed for the intercassette and linker mutants (Fig. 6, *E* and *F*; Table 1). As a result, the NC nucleotide-binding site of the GK1355-6QE mutant had about 2-fold and about 10-fold lower affinities for ADP and ATPγS, respectively, compared with the affinities displayed by the NC pocket of wt hBrr2^T1^. Thus, like the intercassette contacts, nucleotide binding at the CC also gears nucleotide affinities of the NC toward ATP.

### Molecular dynamics simulations suggest intramolecular communication lines that might mediate long-range effects

Our rapid kinetics experiments indicated that the intercassette interface and the linker between NC and CC influence nucleotide binding at both cassettes. Additionally, nucleotide binding to the CC can modulate the kinetics of ADP and ATP interaction with the NC, some 70 Å away. To delineate possible structural communication lines that could mediate these long-range effects, we conducted MD simulations using available crystal structures of apo-hBrr2^T1^ (PDB ID 4F91) and hBrr2^T1^ with ADP bound at the NC and ATP bound at the CC (PDB ID 4F93) (52) to generate models of apo NC-CC (both nucleotide binding pockets empty), NC^ADP^-CC (ADP bound at the NC, CC empty), NC-CC^ATP^ (NC empty, ATP bound at the CC), NC^ADP^-CC^ATP^ (ADP bound at the NC, ATP bound at the CC), and NC^ATP^-CC^ATP^ (ATP bound at either cassette).

**Figure 7:**
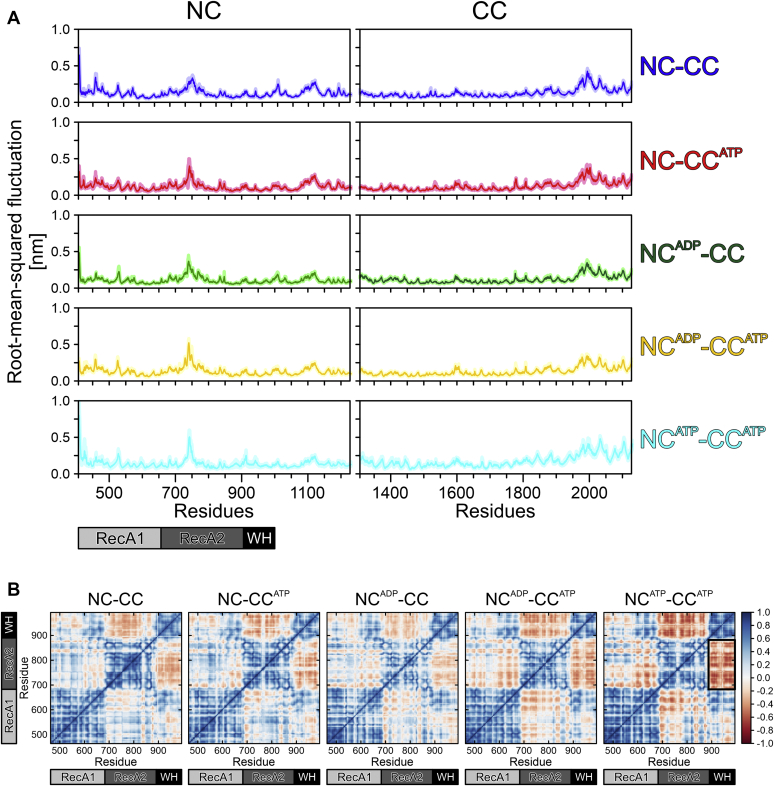
**Brr2 flexibility.***A*, flexibility of the NC (*left*) and of the CC (*right*) computed as root-mean-squared fluctuation. *Solid lines*, mean values; *shaded areas*, SEM, estimated from block averaging of the simulation data (see Experimental procedures). *B*, linear correlation of positional fluctuations of Cα atoms of the RecA1, RecA2, and WH domains in the NC of hBrr2^T1^. Scale bar, degree of linear correlation.

Analysis of positional fluctuations of the Cα-atoms in the MD trajectories revealed no significant differences in empty or nucleotide-bound CCs (Fig. 7*A*, right). In contrast, nucleotide binding to either the NC or the CC led to an increase in the flexibility of the region around residue 750 of the NC (RecA2 domain, region between motifs IV and IVa, involved in RNA binding). Moreover, stronger anticorrelations (darker red regions) in the positional fluctuations between RecA2, RecA1, and in particular the WH domain (Fig. 7*B*) confirm an increased movement in these domains for nucleotide-bound models. The strongest anticorrelation was observed for the NC^ATP^-CC^ATP^ model (Fig. 7*B*, boxed). We refrained from directly comparing flexibilities seen in MD simulations to B-factors of our crystal structures, as the crystal structures contained the hJab1^ΔC^ domain, which was omitted from the MD simulations, and as the B-factors in the crystal structures are modulated by crystal packing, while the MD simulations were done in solution. Together, these observations indicate that nucleotide binding to the NC and CC leads to an additive increase in the flexibility of the region between motifs IV and IVa in the NC RecA2 domain.

**Figure 8:**
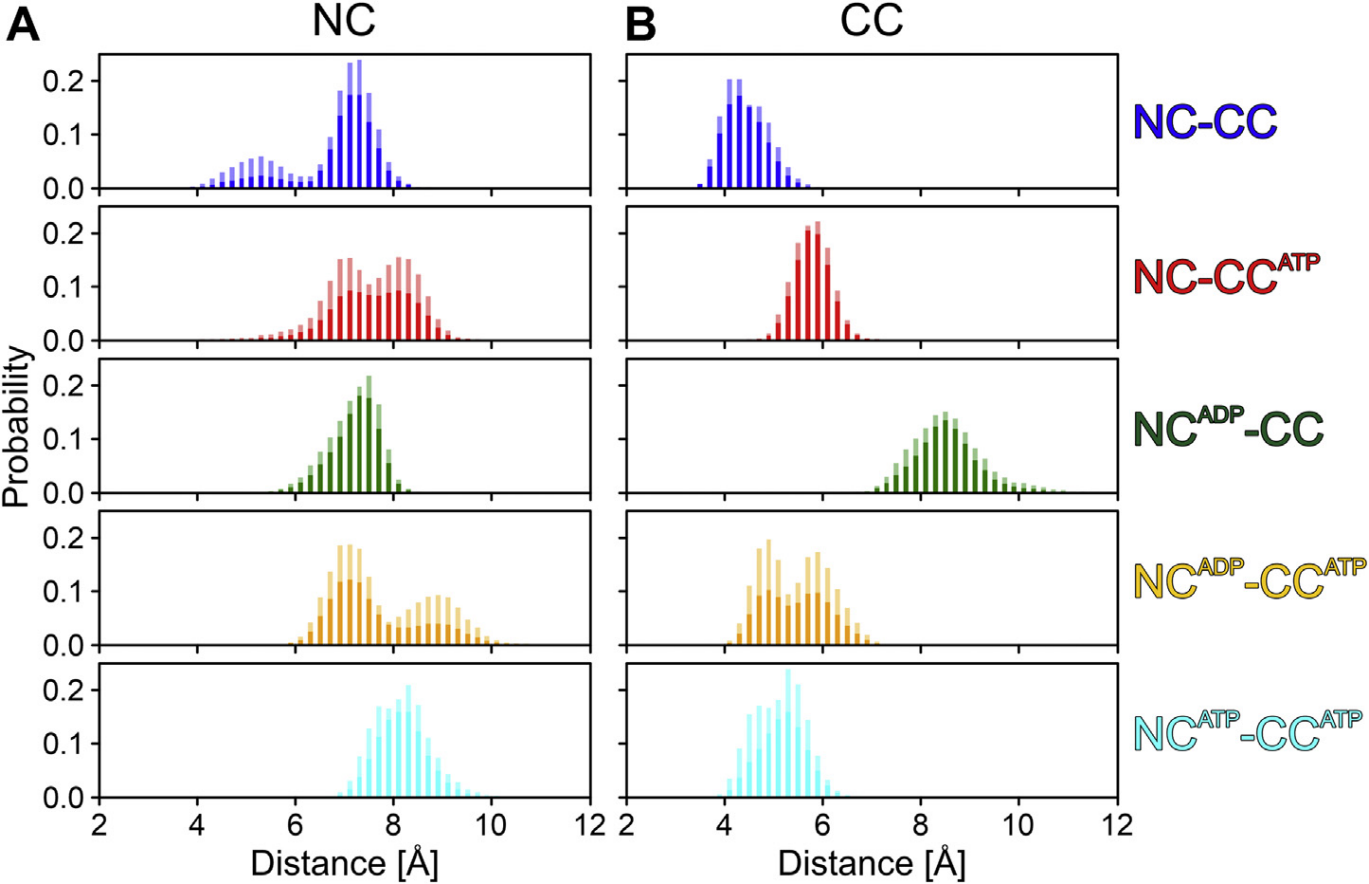
**Widths of the nucleotide-binding pockets.** Widths of the nucleotide-binding pockets of the NC, measured as the distance between G506 and G854 (*left*), and of the CC, measured as the distance between G1353 and G1689 (*right*). *Solid bars*, mean values; *transparent bars*, SEM, represented as mean +SEM.

We next quantified the widths of the nucleotide-binding pockets by the distance distribution between G506 (motif I) and G854 (motif VI, involved in NTP binding/hydrolysis) in the NC as well as between corresponding residues in the CC (G1353 and G1689, respectively; Fig. 8). Nucleotide-bound configurations of both cassettes were generally associated with larger widths of the respective nucleotide-binding pockets compared with the unoccupied states. Moreover, ATP binding at the CC generally induced conformations of the NC associated with wider nucleotide-binding pockets. Likewise, ADP binding at the NC generally led to a wider nucleotide binding pocket at the CC, while ATP at the NC had a small effect in the opposite direction. Irrespective of the detailed effects, these results support the idea of structural communication between the two nucleotide-binding pockets in hBrr2.

To elucidate a possible mechanism of increased flexibilities upon nucleotide binding, hydrogen bond formation in the pockets with and without bound nucleotides was analyzed. In the NC, the average number of hydrogen-bonded contacts between the protein and ADP or ATP was comparable, regardless of the nucleotide bound at the CC (Table S2). In the CC, there were about three more hydrogen bonds between protein and ATP when the NC was also filled compared with the models with an empty NC (see Table S2). Interestingly, one of those additional hydrogen bonds was formed by G1353 (motif I), whose distance to G1689 (motif VI) we used as a measure of the pocket width. Notably, residues binding the nucleotides in the NC or CC did not engage in alternative, intra-hBrr2 hydrogen bonds in the absence of nucleotides. Thus, the observed increase in flexibility of the motif IV-motif IVa region in the NC upon nucleotide binding cannot be explained by redirected hydrogen bonding. However, nucleotide binding at the NC led to additional intraprotein hydrogen bonds between motif I and the Q-motif. In addition to cross-strutting due to the bound nucleotide, such additional intra-hBrr2 hydrogen bonding might explain the increase in melting temperatures observed for all hBrr2 variants upon addition of ATP (Fig. S4). Most notably, N482 formed hydrogen bonds to Q485 when the CC was empty, *i.e.*, to the glutamine of the Q-motif that binds the base of adenine nucleotides. Such sequestration of the Q-motif provides an explanation for our observation of reduced adenine nucleotide binding at the NC in the GK1355-6QE variant (Fig. 6), in which nucleotide binding at the CC is abrogated.

**Figure 9:**
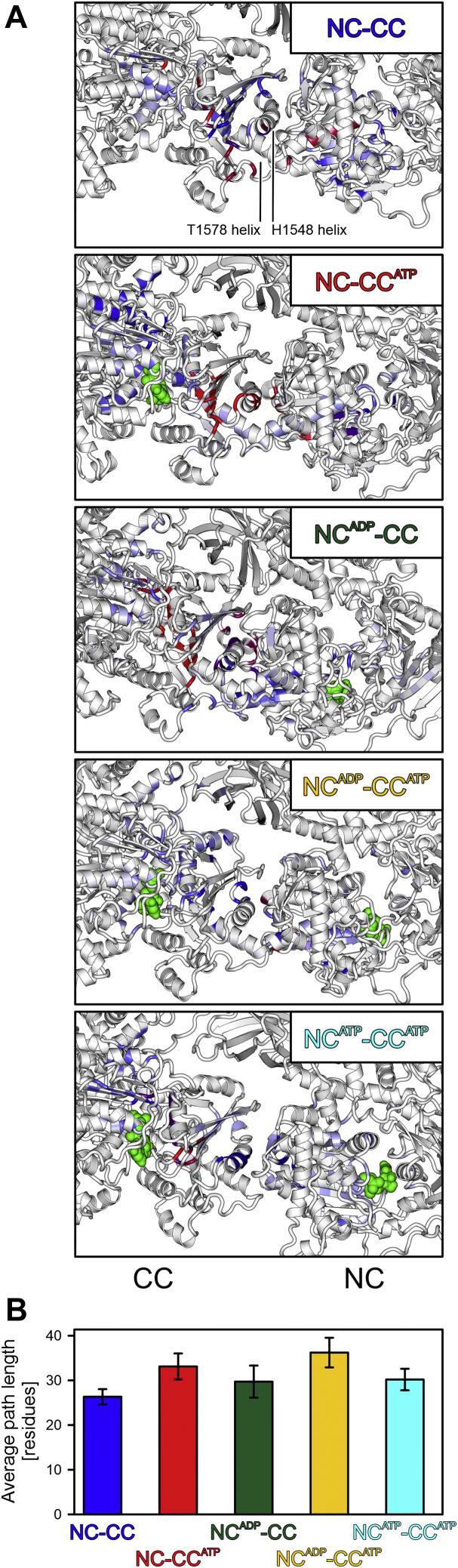
**Shortest paths.***A*, shortest communication paths visualized by highlighting the residues participating in the *k*-shortest pathways between the nucleotide-binding pockets of NC and CC by frequency (low-to-high, *blue-to-red*). *Green spheres*, bound nucleotides. *B*, average path lengths (number of connected residues) of shortest communication paths between the nucleotide-binding pockets of NC and CC. Values represent means ± SEM, with means calculated over five blocks of 20 ns during the last 100 ns of the simulation.

Finally, we sought to investigate the structural basis of long-range communication between the nucleotide-binding pockets of the two cassettes, by which the CC pocket might influence nucleotide binding at the NC and by which residues at the intercassette surfaces might exert their effects on nucleotide binding at either cassette. To this end, we extracted the shortest hydrogen-bonded paths, weighted by hydrogen-bond probabilities between residues along the path, between NC and CC nucleotide-binding pockets from the MD trajectories of the various states (Fig. 9). Details of these shortest paths differed in the different MD trajectories. Model NC^ATP^-CC^ATP^ exhibited the lowest diversity in shortest paths between the pocket residues (Fig. 9*A*). The helix containing T1578 (following motif IV^CC^) was only involved in shortest paths in model NC-CC^ATP^, whereas the helix that contains H1548 (preceding motif IV^CC^) was part of shortest paths connecting the pocket residues in all models except for NC^ADP^-CC^ATP^ (Table 3).

**Table 3:** Participation of mutated residues and of E602 and H1534 in the *k*-shortest paths (*k* = 10)

Path	NC-CC	NC-CC^ATP^	NC^ADP^-CC	NC^ADP^-CC^ATP^	NC^ATP^-CC^ATP^
E616-E1455	R637	E602, R637, K1544, H1548	E602, R637, H1534	R603	E602, K1544, H1548
K509-K1356	R637	E602, K1544, H1548, T1578	E602, H1534	E602, R603	
N820-N1692	R603, H1534	E602, K1544, H1548	E602, R637, H1534	R603	E602, K1544, H1548
Q484-Q1332	E602, R637, H1534	E602, K1544, H1548	E602, H1534, F1717	R603	E602, K1544, H1548
R855-E1455	R603, R637	E602, K1544, H1548	E602, R637, H1534	R603, R637	E578, H1534
T510-T1357	R637	K1544, H1548,T1578	E602, R1195, H1534	R603	E602, K1544, H1548

The hydrogen-bond-based communication between the two cassettes appears to be dominated by the highly probable hydrogen bonds between R603 (between motifs Ic^NC^ and II^NC^) and D1575 (helix following motif IV^CC^), between R637 (between motifs II^NC^ and III^NC^) and D1583 (helix following motif IV^CC^) and between E602 (between motifs Ic^NC^ and II^NC^) and K1544 (preceding motif IV^CC^; Table S3), as these pairs form the majority of the interface crossings (Table S4). It is interesting to note that models with ATP bound to the CC all show shortest paths crossing the interface at a similar location: models NC-CC^ATP^ and NC^ADP^-CC^ATP^ at E602 (to K1544 and subsequently to H1548) and model NC^ADP^-CC^ATP^ at the neighboring R603 (to L1540 or D1575; Table S4).

Together, these observations show that depending on the nucleotide occupation of the cassettes, their intercassette contacts change, providing a molecular basis for the observation that mutations of residues at the cassette interface impact binding of ATPγS to either cassette. The additionally reduced ADP binding to both cassettes upon R603A mutation is furthermore reflected in the importance of R603 in shortest paths of the NC^ADP^-CC^ATP^ model (Table 3).

## Discussion

Results presented here reveal intricate networks of intramolecular communication in the Brr2 RNA helicase that modulate the adenine nucleotide-binding specificities and affinities of the enzyme's N- and C-terminal nucleotide-binding pockets. Changes at intercassette contacts or in a linker connecting the two cassettes affect nucleotide binding at both pockets. Furthermore, the N-terminal nucleotide-binding pocket can “sense” nucleotide binding at the C-terminal pocket, *i.e.*, over a distance of about 70 Å. Brr2 architecture and dynamics suggest likely molecular communication lines that mediate these long-range effects, which run through layers of structural elements in the vicinity of the nucleotide-binding pockets and which connect the two nucleotide-binding pockets across the intercassette interface.

### Use of ATPγS as an ATP surrogate and influence of the mant moieties

Previous studies had shown that ATPγS is not always an ideal ATP surrogate (70) and that some NTPases/helicases can actually employ ATPγS to perform RNA transactions (71). However, we did not observe any significant ATPγS hydrolysis or ATPγS-fueled RNA unwinding activity by hBrr2 *in vitro*. Moreover, our structural analyses suggest that in the case of hBrr2, ATPγS is accommodated in a binding pose as expected for ATP in both nucleotide-binding pockets. Another concern in studies as presented here is that the *mant* moiety, which is indispensable for monitoring nucleotide binding *via* the pre-steady-state kinetics analyses we conducted, might elicit strong effects on nucleotide binding. Again, our crystal structure analyses revealed only very minor alterations in the contacts to the hBrr2 nucleotide-binding pockets fostered by the *mant* moieties of *mant*-ADP or *mant*-ATPγS (Fig. S1). Importantly, the *mant* moieties also did not significantly alter the binding poses of the ADP/ATPγS portions compared with those observed for the unmodified nucleotides (Fig. S1). While we cannot exclude minor differential effects of the *mant* moiety on *mant*-nucleotide binding or dissociation rates for NC and CC, we, therefore, consider it unlikely that such effects caused the much slower dissociation of *mant*-nucleotides from the CC compared with the NC. This notion is supported by a clear structural explanation for slower nucleotide dissociation from the CC, *i.e.*, the apparent locking of nucleotides in the CC by P1694/L1695 (Fig. S3*B*).

### Mechanism of nucleotide binding by Brr2

To date, there are a limited number of reports on kinetic measurements of nucleotide binding to RNA and DNA helicases. To our knowledge, our work represents the first transient kinetics analysis of nucleotide binding to a double-cassette Ski2-like helicase. Due to the presence of two nucleotide-binding pockets in Brr2, we initially characterized the kinetics of *mant*-ADP and *mant*-ATPγS binding and dissociation using single cassette constructs, hBrr2^NC^ and hBrr2^CC^. Detailed characterization of the isolated cassettes allowed us to subsequently unequivocally assign double exponentials observed upon nucleotide binding or dissociation to/from dual-cassette constructs of hBrr2 to either of the cassettes.

*Mant*-nucleotide binding to hBrr2 generates FRET from NC and CC tryptophans to the *mant* moiety of the bound nucleotide. hBrr2 contains one and two tryptophans within about 20 Å distance of nucleotides bound at the NC and CC, respectively (Fig. S2). The differences in the magnitudes of the FRET signals seen with *mant*-ADP or *mant*-ATPγS, with *mant*-ADP binding resulting in a higher amplitude compared with *mant*-ATPγS binding (Fig. 2, *A* and *B*), are explained by lower occupancy of the nucleotide-binding pockets in the case of *mant*-ATPγS, due to a lower affinity of ATPγS compared with ADP (Table 1). Higher affinity for ADP is also reflected in the better defined electron densities for ADP/*mant*-ADP compared with ATPγS/*mant*-ATPγS in the corresponding crystal structures (Fig. 3).

Nucleotide binding to other helicases such as Rep, DnaB, and RecG showed multiphasic kinetics, minimally characterized by a two-step mechanism, with a rapid nucleotide-binding phase followed by nucleotide accommodation that resulted in a state of high nucleotide affinity characterized by high fluorescence (72, 73, 74). For hBrr2, both in the single-cassette and in the double-cassette constructs, nucleotide binding at both pockets could be modeled as a single-step, reversible process, as also observed for the DbpA helicase (75).

### Implications for the differential functions of the two helicase cassettes in Brr2

Our results demonstrate that, in the absence of RNA, *mant*-ATPγS binds more weakly to both cassettes than *mant*-ADP as also observed for the DEAD-box RNA helicase, DbpA (75). In addition, a comparison of the kinetics of nucleotide binding by the hBrr2 cassettes indicates that hBrr2^NC^ binds and releases nucleotides faster than hBrr2^CC^, both in isolation and in the dual-cassette constructs tested (Figs. 4 and 5). The high hBrr2^CC^ nucleotide affinity is the result of a very low nucleotide dissociation rate, with *mant*-ADP and *mant*-ATPγS dissociation being 945 and 800 times slower for hBrr2^CC^ compared with hBrr2^NC^, respectively.

These results are consistent with the functions of the two cassettes for the activity of the enzyme. Fast nucleotide dynamics at the NC are in line with the NC being the active helicase cassette in the protein, which undergoes rounds of nucleotide binding, hydrolysis, and release of the products and which couples these transactions to conformational changes that give rise to translocation of the enzyme on the substrate RNA. The CC, in contrast, is inactive as an enzyme but stimulates the helicase activity of the NC (52). Rather than relying on conformational changes driven by nucleotide transactions, the CC may remain permanently bound to a nucleotide during Brr2-mediated RNA unwinding and provide a stable scaffold, which offers anchoring points for the NC, and can thereby support productive cycles of conformational changes in the NC. This picture is fully in line with our finding that mutations in intercassette contacts and in the intercassette linker, which would in part abrogate the ability of the NC to take advantage of the CC scaffold to transition between conformational states, influence nucleotide binding at the NC (Fig. 6). Furthermore, mutations in the CC nucleotide-binding pocket designed to abrogate nucleotide binding also resulted in reduced nucleotide binding rates at the NC (Fig. 6).

Previously, mutations in the intercassette interface were seen to have only mild effects on the RNA-stimulated ATPase activity of hBrr2 (52). These observations could indicate that, in the presence of RNA, the impact of the mutations on nucleotide binding is not as pronounced as observed in our setup. However, some of the mutations still gave rise to significant defects in the RNA unwinding activity of the respective hBrr2 variants (52). Therefore, we expect that the mutations interfere with the coupling of the ATPase to the helicase activity in the NC.

## Experimental procedures

### Cloning and mutagenesis

Codon-optimized DNA fragments encoding hBrr2^FL^ (residues 1–2136) and fragments thereof (hBrr2^T1^: residues 395–2129; hBrr2^NC^: residues 395–1324; hBrr2^CC^: residues 1282–2136) were cloned into a modified pFL vector (EMBL, Grenoble) to produce proteins with a TEV-cleavable N-terminal His_10_-tag (52) (pFL-His_10_-hBrr2^FL^, pFL-His_10_-hBrr2^T1^, pFL-His_10_-hBrr2^NC^, pFL-His_10_-hBrr2^CC^). Site-directed mutagenesis was performed using the QuikChange II XL Site-Directed Mutagenesis Kit (Stratagene), yielding constructs pFL-His_10_-hBrr2^T1^(R603A), pFL-His_10_-hBrr2^T1^(R637A), pFL-His_10_-hBrr2^T1^(S1087L), pFL-His_10_-hBrr2^T1^(PPP1296-8AAA), pFL-His_10_-hBrr2^T1^(GK1355-6QE), pFL-His_10_-hBrr2^T1^(H1548A). All constructs were verified by sequencing. All plasmids were transformed into *Escherichia coli* DH10MultiBacY cells (provided by Imre Berger, University of Bristol) and further integrated *via* Tn7 transposition into the baculovirus genome (EMBacY) maintained as a bacterial artificial chromosome (BAC) (76). The Tn7 transposition site was embedded in a *lacZα* gene allowing the selection of positive EMBacY recombinants *via* blue/white screening. Recombinant BACs were isolated from the bacterial hosts and used to transfect Sf9 cells (Invitrogen).

### Protein production

All proteins were produced by recombinant baculoviruses in insect cells, as described previously (52, 58). Briefly, for initial virus (V_0_) production, the isolated recombinant EMBacY was transfected into adhesive Sf9 cells (Invitrogen) in 6-well plates. The efficiency of transfection was monitored by eYFP fluorescence. The V_0_ virus generation was used to infect 50 ml Sf9 cells for virus amplification. The second, high-titer virus generation (V_1_) was then used to infect 1200 ml High Five cells (Invitrogen) for large-scale protein production. The infected cells were harvested when the eYFP signal reached a plateau and before the cell viability dropped below 90%.

### Protein purification

Proteins were purified as described previously (52, 58). Briefly, for protein production for biochemical/biophysical experiments, the High Five cell pellet was resuspended in 50 mM HEPES-NaOH, pH 8.0, 600 mM NaCl, 2 mM β-mercaptoethanol, 0.05% NP40, 1.5 mM MgCl_2_, 20 (v/v) % glycerol, 10 mM imidazole, supplemented with EDTA-free protease inhibitor (Roche) and lyzed by sonication using a Sonopuls Ultrasonic Homogenizer HD 3100 (Bandelin). The target was captured from the cleared lysate on a 5 ml HisTrap FF column (GE Healthcare) and eluted with a linear gradient from 10 to 250 mM imidazole. The eluted protein containing the protein of interest was diluted to a final concentration of 80 mM NaCl, treated with RNaseA (Sigma), and loaded on a Mono Q 10/100Gl column (GE Healthcare) equilibrated with 50 mM Tris-HCl, pH 8.0, 50 mM NaCl, 5 (v/v) % glycerol, 2 mM β-mercaptoethanol. The protein was eluted with a linear 0.05–1.5 M NaCl gradient and further purified by gel filtration on a HiLoad Superdex 200 16/60 column (GE Healthcare) in 40 mM Tris-HCl, pH 8.0, 200 mM NaCl, 20 (v/v) % glycerol, 2 mM DTT. The peak fractions were concentrated, flash-frozen in liquid nitrogen, and stored at –80 °C.

For protein production for crystallization, the hBrr2^T1^ insect cell pellet was lysed by sonification for 30 min in 50 mM HEPES-NaOH pH 7.5, 600 mM NaCl, 10% (w/v) glycerol, 0.05% (v/v) Nonidet P-40, 20 μg/ml DNase I, 2 mM β-mercaptoethanol, containing Complete EDTA-free protease inhibitors. After centrifugation and loading onto a HisTrap FF column, the protein was eluted with 250 mM imidazole. TEV protease was added for cleavage of the His-tag, and the mixture was dialyzed overnight in 40 mM HEPES-NaOH, pH 7.5, 500 mM NaCl, 10% (w/v) glycerol, 15 mM imidazole, 2 mM β-mercaptoethanol. The cleaved protein was collected in the flow-through of a HisTrap FF column. After fivefold dilution with 25 mM Tris-HCl, pH 8.0, 50 mM NaCl, 5% (v/v) glycerol, 2 mM DTT and treatment with RNase A, the protein was loaded on a HiPrep Heparin FF column (GE Healthcare) equilibrated with 25 mM Tris-HCl, pH 8.0, 50 mM NaCl, 5% (v/v) glycerol, 2 mM DTT, and eluted by a linear increase of NaCl to 750 mM. The fractions of interest were combined and chromatographed on a HiLoad Superdex 200 16/60 column in 10 mM Tris-HCl, pH 7.5, 200 mM NaCl, concentrated to 10 mg/ml, flash-frozen in liquid nitrogen, and stored at –80 °C.

The hJab1^ΔC^ insect cell pellet was lysed by sonification for 30 min in 50 mM Tris-HCl, pH 8.0, 300 mM NaCl, 5% (v/v) glycerol, 0.05% (v/v) NP-40, 2 mM DTT, supplemented with Complete EDTA-free protease inhibitors. After centrifugation, the protein was captured on glutathione sepharose beads (GE Healthcare) and eluted with 10 mM reduced glutathione. Buffer was exchanged to 50 mM Tris-HCl, pH 8.0, 300 mM NaCl, 5% (v/v) glycerol, 2 mM DTT on a HiLoad Superdex 75 26/60 column (GE Healthcare). After treatment with Prescission protease overnight, the hJab1^ΔC^ protein lacking the GST tag was collected in the flow-through of glutathione sepharose beads. Subsequently, the protein was loaded on a HiLoad Superdex 75 16/60 column in 10 mM Tris-HCl, pH 8.0, 150 mM NaCl, concentrated to 4 mg/ml, flash-frozen in liquid nitrogen, and stored at –80 °C.

For complex formation, hBrr2^T1^ was combined with a 1.5-fold molar excess of hJab1^ΔC^, and the complex was purified by gel filtration on a Superdex 200 10/300 global increase column (GE Healthcare) in 20 mM Tris-HCl, pH 8.0, 150 mM NaCl, concentrated to 6 mg/ml, flash-frozen in liquid nitrogen, and stored at –80 °C.

### Crystallographic analyses

Crystals of the hBrr2^T1^-hJab1^ΔC^ complex were grown in a 24-well plate with a reservoir solution of 0.1 M HEPES-NaOH, pH 8.0, 0.1 M MgCl_2_, 8% PEG 3350, as described before (58). Crystals were soaked for 1 h in 10 mM ADP or ATPγS, or in 1 mM *mant*-ADP or *mant*-ATPγS (Jena Bioscience) in reservoir solution. After cryoprotection with 25% (v/v) ethylene glycol in reservoir solution, crystals were flash-cooled in liquid nitrogen.

Diffraction data were acquired at beamline 14.2 of the BESSY II storage ring (Berlin, Germany) and processed with XDS (77). Molecular replacement was done with Phenix (78) using the structure coordinates of the hBrr2^T1^-hJab1^ΔC^ complex (PDB ID 6S8Q) (58). The structures were manually adjusted with Coot (79) and automatically refined with Phenix. Restraints for the ligands were generated by the “eLBOW”-tool of Phenix. Structure figures were prepared with PyMOL (Version 1.8 Schrödinger, LLC).

### Characterization of protein variants

DSF experiments were done in a 96-well plate in a plate reader combined with a thermocycler (Stratagene M x 3005P). All hBrr2 constructs were diluted to a final concentration of 3 μM in purification buffer (±2 mM ATP/Mg^+2^) supplemented with 10×SYPRO orange (1:500 dilution of the stock) in a total volume of 20 μl. The temperature was increased linearly from 25 °C to 95 °C, and the fluorescence emission was monitored in steps of 1 °C/min with hold steps of 30 s between reads. The fluorescence intensity was then plotted as a function of temperature. The sigmoidal curve from each construct was normalized and corrected for the background signal of the fluorophore in the buffer. The inflection points of the curves, representing the thermal melting temperature, were extracted from the first derivatives of the melting profiles using OriginLab. Each measurement was performed once.

### Rapid kinetic measurements

The kinetics of the interaction of hBrr2 variants with nucleotides were characterized *via* fluorescence stopped-flow measurements on an SX-20MV spectrometer (Applied Photophysics). The fluorescence of *mant*-labeled nucleotides was excited using 290 nm light *via* FRET from tryptophan residues in the proximity of the nucleotide-binding pockets and measured at 90° after passing a cutoff filter (KV 408, Schott). FRET was observed only when both donor and acceptor were present since a negligible fluorescence change of tryptophan was observed when nonfluorescent nucleotides were bound to the proteins. Association experiments were performed by rapidly mixing equal volumes (60 μl) of the reactants (syringe 1 contained hBrr2 variants while syringe 2 contained the *mant* nucleotides) in 20 mM HEPES-NaOH, pH 8.0, 150 mM NaCl, 1.5 mM MgCl_2_ at 20 °C and monitoring fluorescence change over time. Dissociation or chase experiments were performed similarly by rapidly mixing equal volumes (60 μl) of the reactants (syringe 1 contained hBrr2 variants in complex with *mant* nucleotides while syringe 2 contained an excess of unlabeled nucleotides) at 20 °C and monitoring fluorescence change over time. In all cases, 1000 data points were acquired in logarithmic sampling mode. The data were visualized using the Pro-Data Viewer software package (Applied Photophysics). The final curves were obtained by averaging seven to ten individual traces after normalizing each data point to initial F_0_. Data were evaluated by fitting to a single exponential function with a characteristic time constant (*k*_*app*_), amplitude (*F*_1_), and another variable for the final signal (*F*_*∞*_) according to the equation, *F* = *F*_*∞*_ + *F*_1_exp(-*k*_*app*_t), in which *F* is the fluorescence at time *t*. For constructs containing two nucleotide-binding sites, two exponential terms were used with two characteristic time constants (*k*_*app*1_, *k*_*app*2_), amplitudes of the signal change (*F*_1_, *F*_2_), and another variable for the final signal (*F*_*∞*_) according to the equation, *F* = *F*_*∞*_ + *F*_1_exp(-*k*_*app*1_t) + *F*_2_exp(-*k*_*app*2_t). Dependencies of the apparent rate constants on nucleotide concentration were fitted by a linear equation, *k*_*app*_ = *k*_1_[mant-nucleotide]+*k*_−1_, in which *k*_1_ represents the nucleotide association rate constant (derived from the slope), and *k*_−1_ represents the nucleotide dissociation rate constant (derived from the Y-axis intercept). Calculations and statistical analysis were performed using Prism software (GraphPad). Single exponential fitting to the single-cassette hBrr2 constructs or double exponential fitting to the double-cassette hBrr2 constructs resulted in the best fits (lowest SEMs) of the experimental data and the best 95% confidence intervals, as evaluated by Prism software.

### Molecular dynamics simulations

hBrr2^403–2125^ was modeled based on PDB entries 4F91 (for the apo form) and 4F93 (for nucleotide-bound forms) (52). ADP was changed *in silico* to ATP, and one magnesium ion was modeled into the NC-binding pocket using its position relative to ATP in the CC pocket as a template. Proteins, nucleotides, and ions were described with the CHARMM force field (80). The (nucleotide-bound) proteins were first relaxed by 500 steps of steepest descend. The relaxed structures were then solvated by TIP3P water (81) in a dodecahedral box, extending 1.5 nm from the solute (~1.8 nm length). After 5000 steps of optimization, keeping the nucleotide fixed, 1 ns of MD simulations with all protein heavy atoms positionally restrained were performed to further equilibrate the systems at 300 K, using a Berendsen thermostat (82). Finally, MD production runs of 200 ns length were performed in an NVT ensemble, at 300 K controlling temperature by canonical sampling through canonical velocity rescaling (83), and 2 fs time steps for the integration with the LINCS algorithm (84) to constrain covalent bonds. Electrostatic interactions were treated with the particle mesh Ewald method (85) on a grid with 0.16 spacing and a short-range cutoff of 1.4 nm. The same cutoff was applied to the van der Waals interactions. All simulations were performed with Gromacs 4.6.7 (86).

For the analyses, only the last 100 ns simulation time was considered. Hydrogen bonds were defined geometrically by a 3.2 Å maximal distance between donor (D) and acceptor (A) atoms and 42° maximum deviation from linearity for the D-H···A angle.

For the communication analysis, a weighted graph was constructed, in which the protein residues form the nodes and edges between nodes were defined by the occurrence of a hydrogen bond in the course of the simulation. The probabilities of these hydrogen bonds to occur served as edge weights. The probability of hydrogen bonds between two residues i and j, HB_ij_, was converted into communication costs C_ij_ = −ln(HB_ij_). Consecutive residues, *i.e.*, covalently bound, were assigned a communication cost of zero. Shortest paths were selected from the resulting communication graph using Dijkstra's algorithm (87), with the path lengths taken as the sum of the edge weights along the path. The analysis of hydrogen bonds, setup, and evaluation of communication graphs were carried out using our own Java code, based on the Jgraph library (88).

Protein flexibilities were analyzed using the rmsf tool of the Gromacs program. Errors were estimated from block averaging, partitioning the last 100 ns of the simulation data into five blocks of 20 ns length each.

### Data availability

Coordinates and structure factors for crystal structures presented in this article have been deposited in the RSCB Protein Data Bank (https://www.rcsb.org) under the following accession codes: 7BDI (hBrr2^T1^-hJab1^ΔC^-ATPγS; https://doi.org/10.2210/pdb7BDI/pdb), 7BDJ (hBrr2^T1^-hJab1^ΔC^-*mant*-ATPγS; https://doi.org/10.2210/pdb7BDJ/pdb), 7BDK (hBrr2^T1^-hJab1^ΔC^-ADP; https://doi.org/10.2210/pdb7BDK/pdb) and 7BDL (hBrr2^T1^-hJab1^ΔC^-*mant*-ADP; https://doi.org/10.2210/pdb7BDL/pdb; Table S1). All other data are contained within the article.

## Supporting information

This article contains supporting information (89).

## Conflict of interest

The authors declare that they have no conflicts of interest with the contents of this article.
