# Supplementary material for: Long-range allostery mediates cooperative adenine nucleotide binding by the Ski2-like RNA helicase Brr2
Source: J Biol Chem. 2021 May 26;297(1):100829. doi: 10.1016/j.jbc.2021.100829 (PMC8220420; doi:10.1016/j.jbc.2021.100829)
Supplement: Tables S1–S4 and Figures S1–S4 [file mmc1.docx]

**Supplemental material**

**Long-range allostery mediates cooperative adenine nucleotide binding by the Ski2-like RNA helicase Brr2**

Eva Absmeier^1,†,∞^, Karen Vester^1,†^, Tahereh Ghane^2^, Dmitry Burakovskiy^3,$^, Pohl Milon^4^, Petra Imhof^2,¶^, Marina V. Rodnina^3^, Karine F. Santos^1,†,#,^*, Markus C. Wahl^1,5,^*

From the ^1^Freie Universität Berlin, Structural Biochemistry, Takustr. 6, D-14195 Berlin, Germany; ^2^Freie Universität Berlin, Computational Biophysics, Arnimallee 14, D-14195 Berlin, Germany; ^3^Max Planck Institute for Biophysical Chemistry, Department of Physical Biochemistry, Am Faßberg 11, D-37077 Göttingen, Germany; ^4^Centre for Research and Innovation, Health Sciences Faculty, Universidad Peruana de Ciencias Aplicadas, Lima, 15023, Perú; ^5^Helmholtz-Zentrum Berlin für Materialien und Energie, Macromolecular Crystallography, Albert-Einstein-Straße 15, D-12489 Berlin, Germany

^†^ These authors contributed equally to this work.

^∞^ Present address: MRC Laboratory of Molecular Biology, Francis Crick Avenue, Cambridge CB2 0QH, UK

^$^ Present address: Evotec SE, Essener Bogen 7, D-22419 Hamburg, Germany

^¶^ Present address: Friedrich-Alexander Universität Erlangen-Nürnberg, Department of Chemistry and Pharmacy, Computational Chemistry Center, Nägelsbachstr. 25, D-91052 Erlangen, Germany

^#^ Present address: 4TEEN4 Pharmaceuticals GmbH, Neuendorfstr. 15b, D-16761 Hennigsdorf, Germany

* To whom correspondence should be addressed: Karine F. Santos Bourgeois: 4TEEN4 Pharmaceuticals GmbH, Neuendorfstr. 15b, D-16761 Hennigsdorf, Germany; bourgeois@4teen4.de; Markus C. Wahl: Freie Universität Berlin, Structural Biochemistry, Takustr. 6, D-14195 Berlin, Germany; mwahl@zedat.fu-berlin.de; Tel.: +49-30-838-53456; Fax: +49-30-8384-53456

Running title: Long-range allosteric effects in an RNA helicase

**Keywords:** Allosteric regulation, enzyme kinetics, intra-molecular regulation, pre-mRNA splicing, protein conformation, RNA helicase, superfamily 2 helicase

**Supplemental TABLES**

**Table S1.** Crystallographic data.

| **Dataset** | **hBrr2^T1^-**  **hJab1^ΔC^-**  **ATPγS** | **hBrr2^T1^-**  **hJab1^ΔC^-**  ***mant*-ATPγS** | **hBrr2^T1^-**  **hJab1^ΔC^-**  **ADP** | **hBrr2^T1^-**  **hJab1^ΔC^-**  ***mant*-ADP** |
| --- | --- | --- | --- | --- |
| **Data Collection** | | | | |
| **Wavelength** [Å] | 0.9184 | 0.9184 | 0.9184 | 0.9184 |
| **Temperature** [K] | 100 | 100 | 100 | 100 |
| **Space group** | P2_1_2_1_2_1_ | P2_1_2_1_2_1_ | P2_1_2_1_2_1_ | P2_1_2_1_2_1_ |
| **Unit cell parameters** [Å]  a, b, c | 99.7, 118.7, 187.2 | 99.6, 118.9, 187.4 | 99.5, 118.6, 187.2 | 99.9, 118.8, 187.8 |
| **Resolution** [Å]^a^ | 50.0-2.8  (2.97-2.80) | 50.0-2.6  (2.75-2.60) | 50.0-2.5  (2.67-2.50) | 50.0-2.7  (2.86-2.70) |
| **Reflections**  Unique  Completeness [%]  Redundancy | 55,396 (8,662)  99.5 (97.8)  6.7(6.7) | 69,284 (10,681)  99.3 (96.1)  6.6(6.6) | 74,606 (11,542)  98.6 (95.6)  6.7(6.7) | 62,315 (9,697)  99.5 (97.2)  6.7(6.7) |
| **I/σ(I)** | 6.5 (1.0) | 8.96 (1.0) | 7.40 (1.0) | 7.47 (1.0) |
| **R_meas_(I)** [%]^b^ | 31.9 (222.8) | 20.8 (212.9) | 26.3 (210.5) | 25.1 (185.4) |
| **CC_1/2_** [%]^c^ | 99.0 (38.8) | 99.7 (40.0) | 99.3 (35.6) | 99.4 (40.4) |
| **Refinement** | | | | |
| **Resolution** [Å]^a^ | 48.2-2.8  (2.9-2.8) | 46.8-2.6  (2.69-2.59) | 46.8 -2.5  (2.61-2.52) | 48.3-2.7  (2.79-2.69) |
| - - - - 1. **Reflections**   Number  Completeness [%]  Test set | 55,337 (5,424)  99.83 (99.43)  2,099 (206) | 69,279 (6,469)  99.28 (93.82)  2100 (196) | 74,594 (7053)  98.80 (94.68)  2,100 (198) | 62,304 (5,860)  99.45 (95.47)  2,100 (197) |
| **R_work_**^d^ | 23.7 (35.4) | 23.0 (33.7) | 22.8 (33.2) | 23.2 (34.1) |
| **R_free_**^e^ | 29.7 (38.4) | 28.9 (40.2) | 28.4 (35.9) | 29.2 (42.6) |
| **Contents of A.U.**^f^  Protein atoms  Ligand atoms  Water oxygens | 15,988  64  217 | 15,965  84  148 | 15,884  56  309 | 15,988  76  292 |
| **Mean B factors** [Å^2^]   - - - - 1. Wilson   Protein  Ligands  Water oxygens | 55.6  57.0  48.9  42.0 | 53.7  65.3  79.5  49.8 | 46.8  55.7  46.6  45.7 | 53.0  60.3  58.9  43.1 |
| **Ramachandran plot**^g^  Favored [%]  Outliers [%] | 93.44  1.11 | 92.87  0.71 | 95.42  0.46 | 93.8  0.81 |
| **Rmsd**^h^  Bond lengths [Å]  Bond angles [°] | 0.006  0.58 | 0.003  0.64 | 0.006  0.67 | 0.003  0.53 |
| **PDB ID** | 7BDI | 7BDJ | 7BDK | 7BDL |

^a^ Values in parentheses refer to the highest resolution shells.

^b^ R_meas_(I) = ∑_h_ [N/(N-1)]^1/2^ ∑_i_ │I*_i_*_h_ - <I_h_>│ / ∑_h_∑_i_ I*_i_*_h_, in which <I_h_> is the mean intensity of symmetry-equivalent reflections h, I*_i_*_h_ is the intensity of a particular observation of h and N is the number of redundant observations of reflection h.

^c^ CC_1/2_ = (<I^2^> - <I>^2^) / (<I^2^> - <I>^2^) + σ^2^_ε_, in which σ^2^_ε_ is the mean error within a half-dataset (89).

^d^ R_work_ = ∑_h_ │F_o_ - F_c_│ / ∑ F_o_ (working set, no σ cut-off applied).

^e^ R_free_ is the same as R_work_, but calculated on the test set of reflections excluded from refinement.

^f^ A.U. – asymmetric unit.

^g^ Calculated with Phenix.

^h^ Rmsd - root-mean-square deviation from target geometry.

**Table S2.** Number of hydrogen bonds between protein and nucleotides.

| **Cassette** | **NC-CC^ATP^** | **NC^ADP^-CC** | **NC^ADP^-CC^ATP^** | **NC^ATP^-CC^ATP^** |
| --- | --- | --- | --- | --- |
| **NC** |  | 7.8±0.8 | 7.0±1.3 | 7.5±2.7 |
| **CC** | 4.0 ±0.9 |  | 7.1±1.1 | 6.8±1.1 |

**Table S3.** Hydrogen bond occupancies between the two cassettes [%].

| **Hydrogen bond** | **NC-CC** | **NC-CC^ATP^** | **NC^ADP^-CC** | **NC^ADP^-CC^ATP^** | **NC^ATP^-CC^ATP^** |
| --- | --- | --- | --- | --- | --- |
| **E602-T1537** | 71.3±5.5 |  |  |  |  |
| **E602-K1544** | 100.0±2.9 | 81.0±38.9 |  | 100.0±3.0 |  |
| **R603-*L1540*^a^** |  | 52.1±29.8 | 60.9±9.3 |  |  |
| **R603-D1575** | 100.0±15.5 | 100.0±2.4 | 100.0±0.6 |  | 100.0±25.8 |
| **R637-D1583** |  | 100.0±42.8 | 68.3±75.9 | 100.0±11.7 | 100.0±15.7 |
| **N909-K1544** |  | 52.8±27.5 |  | 74.2±7.5 | 67.1±14.9 |
| **N909-H1548** |  |  | 48.0±8.9 |  |  |
| **Q1191-*Y1770*** |  | 47.7±19.1 |  |  |  |
| ***P1192-N1767*** | 64.8±9.1 | 49.9±15.8 | 65.6±20.2 | 52.9±7.2 |  |
| **S1196-K1716** | 51.3±20.6 |  |  |  |  |
| **E1237-S1709** |  |  |  | 42.8±22.8 |  |

^a^ Hydrogen bonds of the backbone in italics.

**Table S4.** Crossing of cassette-cassette interface on shortest paths.

| **Path** | **NC-CC** | **NC-CC^ATP^** | **NC^ADP^-CC** | **NC^ADP^-CC^ATP^** | **NC^ATP^-CC^ATP^** |
| --- | --- | --- | --- | --- | --- |
| **E616-E1455** | R637-D1583 | E602-K1544 | R637-D1583 | R603-L1540 | E602-K1544 |
| **K509-K1356** | R637-D1583 | E602-K1544 | Y605-H1534 | R603-D1575 | Q980-Q1528 |
| **N820-N1692** | K599-H1534 | E602-K1544 | Y605-H1534 | R603-L1540 | E602-K1544 |
| **Q484-Q1332** | E578-H1534 | E602-K1544 | Y605-H1534 | R603-D1575 | E602-K1544 |
| **R855-E1455** | R637-D1583 | E602-K1544 | R637-D1583 | R603-L1540 | E578-H1534 |
| **T510-T1357** | R637-D1583 | M641-T1578 | Y605-H1534 | R603-D1575 | E602-K1544 |

**SUPPLEMENTAl FIGURES**


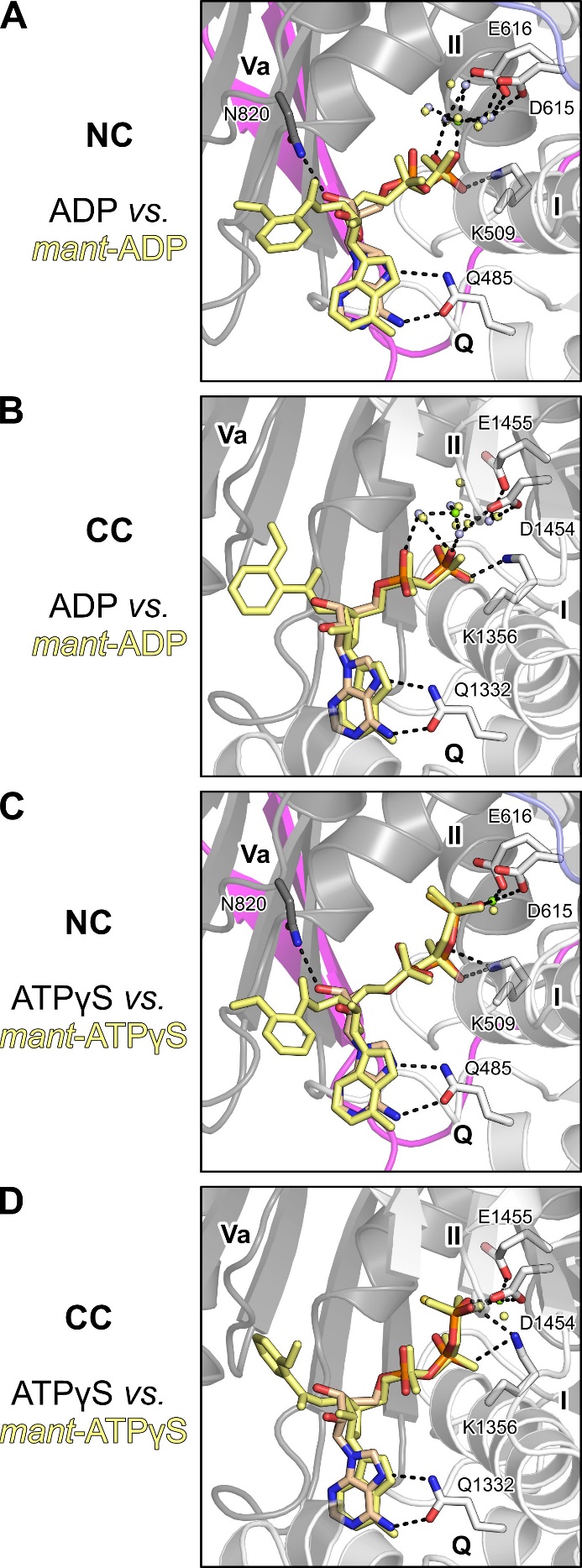


**Figure S1. Comparison of nucleotide binding poses.** **A**. Comparison of ADP and *mant*-ADP binding to the NC. **B**. Comparison of ADP and *mant*-ADP binding to the CC. **C**. Comparison of ATPγS and *mant*- ATPγS binding to the NC. **D**. Comparison of ATPγS and *mant*-ATPγS binding to the CC. Structures of the various nucleotide-bound states were super-imposed by global spatial alignment of the protein parts (hBrr2^T1^-hJab1^ΔC^). Only the hBrr2^T1^-hJab1^ΔC^ scaffold of the complexes with the unmodified nucleotides are shown. *Mant*-nucleotides, pale yellow. Other coloring as in Fig. 1B and Fig. 3.


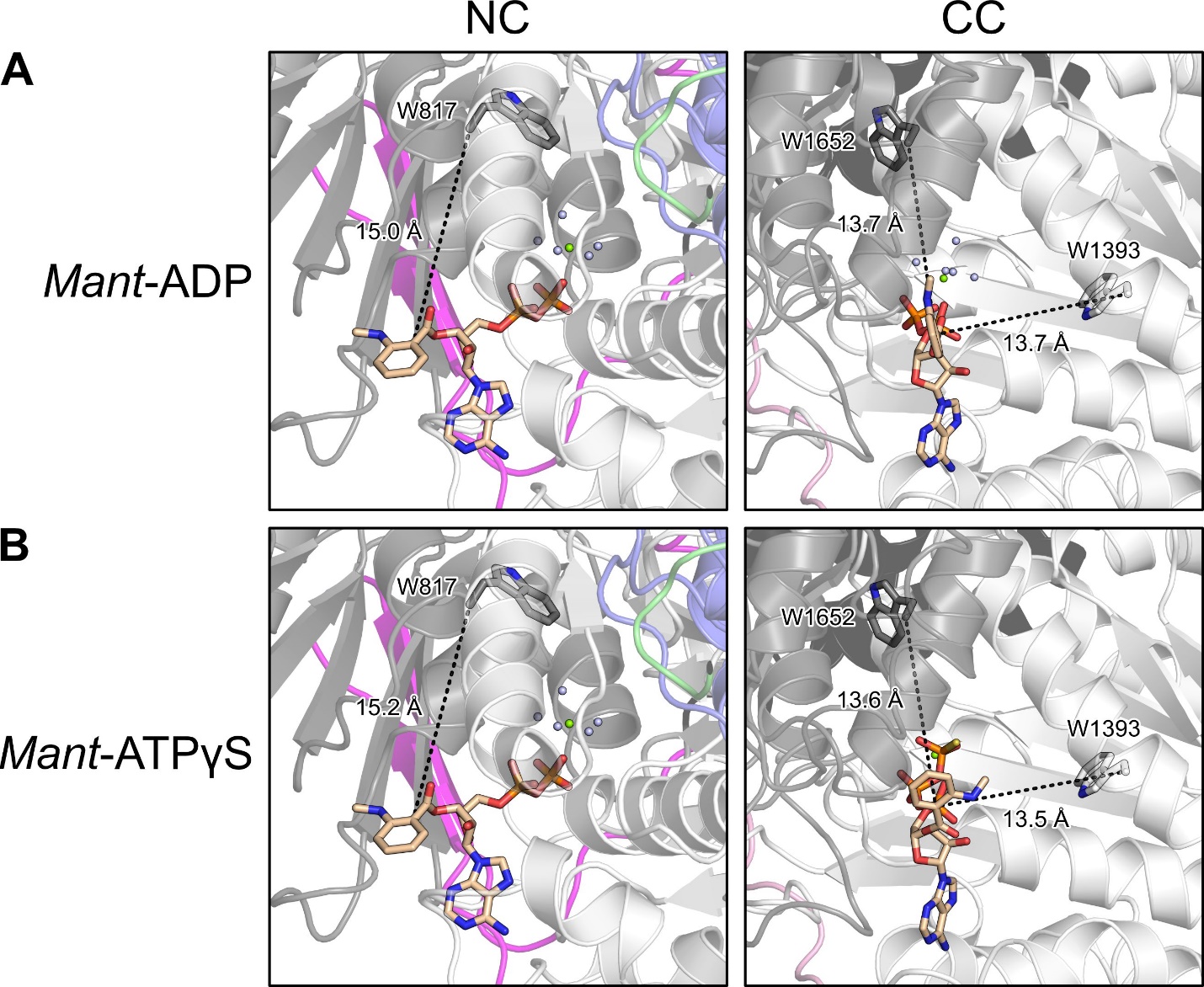


**Figure S2. Distances of Trp residues to *mant* moieties.** **A** and **B**. Comparison of the distances of the *mant* moieties in *mant*-ADP (**A**) or *mant*-ATPγS (**B**) to the nearest Trp residues around the NC (left) and CC (right) nucleotide binding pockets. Dashed lines, distances between the C8 atom of the *mant*-nucleotides to the Cα atoms of the respective Trp residues.


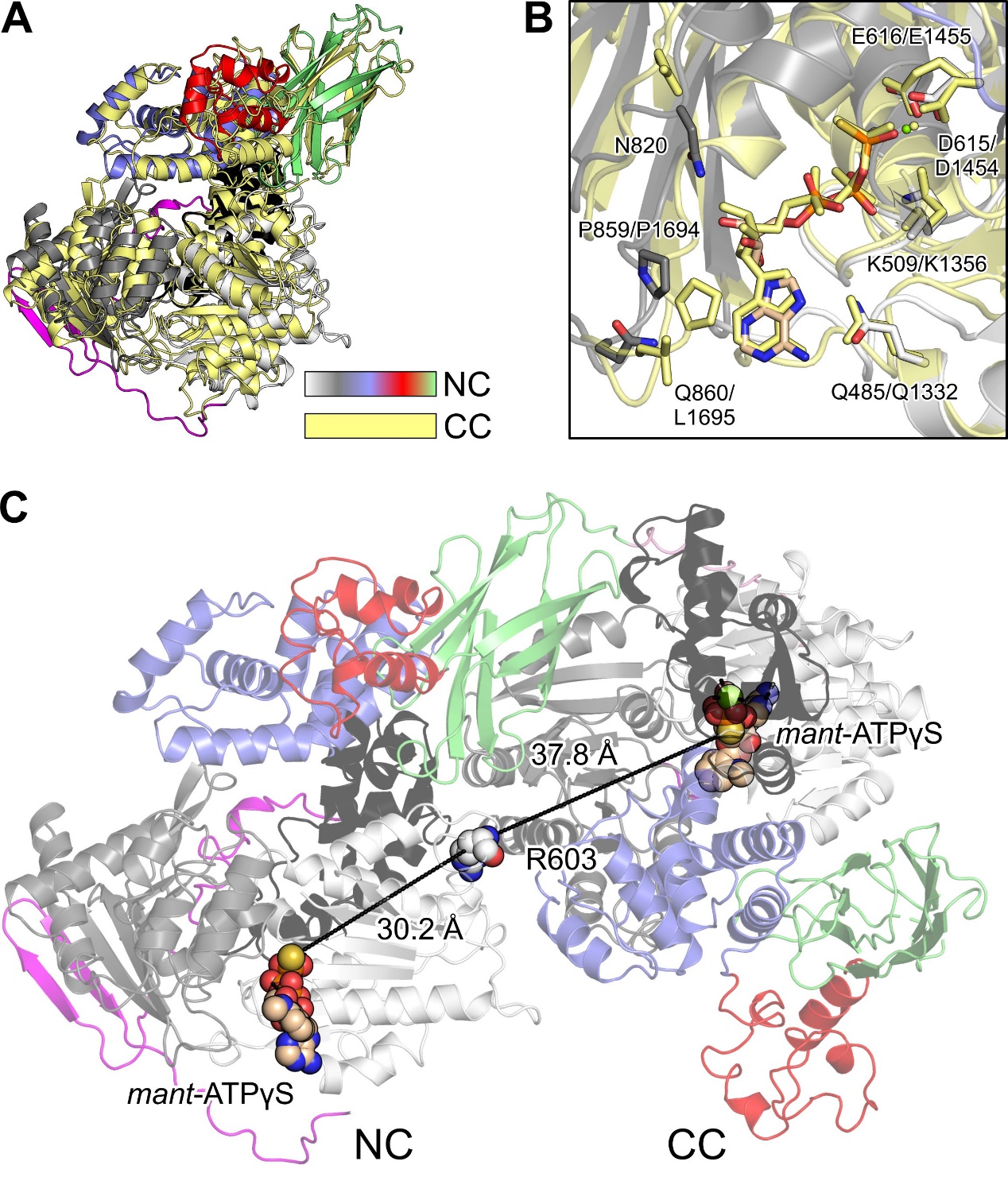


**Figure S3. Structural comparison of NC and CC and position of R603.** **A**. Global super-positioning of the CC (pale yellow) onto the NC (colored by domains as in Fig. 1B), showing similar overall structures of the two helicase cassettes (based on the ATPγS-bound structure of hBrr2^T1^-hJab1^ΔC^ as an example). The view of the NC is identical to Fig. 1B. **B**. Comparison of the NC and CC nucleotide binding pockets after super-positioning of the CC (pale yellow) onto the NC (colored by domain as in Fig. 1B) according to the RecA1 domains (based on the ATPγS-bound structure of hBrr2^T1^-hJab1^ΔC^ as an example). **C**. Position of R603 at the inter-cassette interface relative to the NC (left) and CC (right) nucleotide binding pockets. The *mant*-ATPγS-bound structure of hBrr2^T1^-hJab1^ΔC^ was used for display (hJab1^ΔC^ omitted). Black lines, distances between the R603 Cα atom and the γ-thiophosphate moieties of the *mant*-ATPγS molecules.


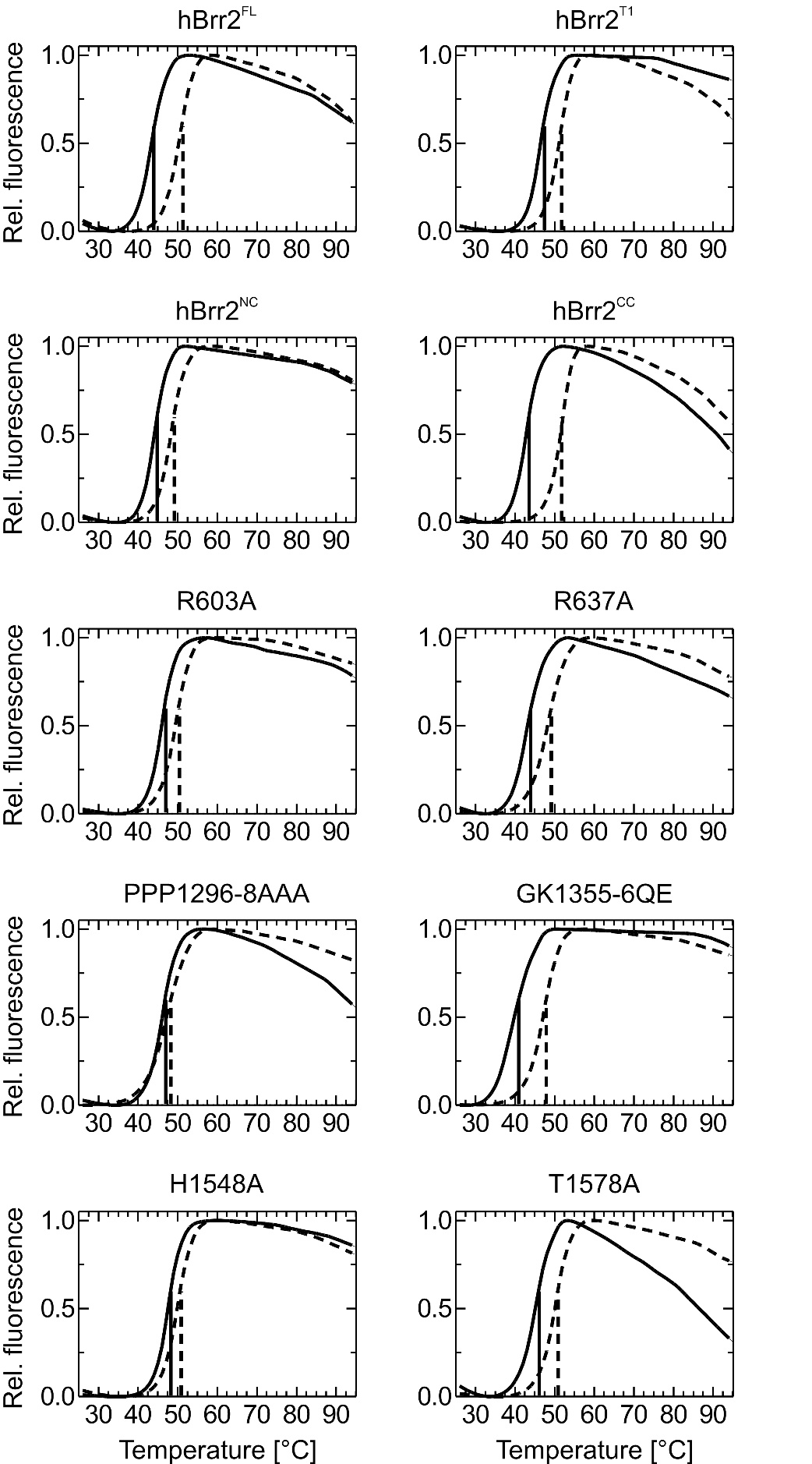


**Figure S4. DSF analyses of the hBrr2 variants used in this work.** Solid lines, melting curves obtained in the absence of ATP/Mg^+2^; dashed lines, melting curves obtained in the presence of 2 mM ATP/Mg^+2^. Each curve resulted from a single measurement. All variants exhibited cooperative transitions with similar melting temperatures.
